# Unravelling the Complexity of Human Olfactory Receptor Repertoire by Copy Number Analysis across Population Using High Resolution Arrays

**DOI:** 10.1371/journal.pone.0066843

**Published:** 2013-07-03

**Authors:** Avinash M. Veerappa, Sangeetha Vishweswaraiah, Kusuma Lingaiah, Megha Murthy, Dinesh S. Manjegowda, Radhika Nayaka, Nallur B. Ramachandra

**Affiliations:** 1 Genomics Lab, Department of Studies in Zoology, University of Mysore, Manasagangotri, Mysore, India; 2 Department of Anatomy, Yenepoya Medical College, Yenepoya University, Mangalore, India; Institut Jacques Monod, France

## Abstract

Olfactory receptors (OR), responsible for detection of odor molecules, belong to the largest family of genes and are highly polymorphic in nature having distinct polymorphisms associated with specific regions around the globe. Since there are no reports on the presence of copy number variations in OR repertoire of Indian population, the present investigation in 43 Indians along with 270 HapMap and 31 Tibetan samples was undertaken to study genome variability and evolution. Analysis was performed using Affymetrix Genome-Wide Human SNP Array 6.0 chip, Affymterix CytoScan^®^ High-Density array, HD-CNV, and MAFFT program. We observed a total of 1527 OR genes in 503 CNV events from 81.3% of the study group, which includes 67.6% duplications and 32.4% deletions encompassing more of genes than pseudogenes. We report human genotypic variation in functional OR repertoire size across populations and it was found that the combinatorial effect of both “orthologous obtained from closely related species” and “paralogous derived sequences” provide the complexity to the continuously occurring OR CNVs.

## Introduction

Olfactory receptors (OR) belong to the hyperfamily of seven-helix G-protein-coupled receptors (GPCRs), involved in conversion of the odorant into odor sensation [1]. This sense of smell includes a cascade of biochemical and electrophysiological processes to detect and discriminate millions of odorous compounds [2]. These olfactory receptors constitute the largest mammalian multi-gene family organized in 40 clusters across 21 chromosomes [3]. Such a cluster organization is the result of extensive processes of expansion, diversification, duplication, deletion and pseudogenization [4]. This process of OR creation and annihilation by genomic rearrangements is ratified by prevailing Copy Number Variations (CNVs) [1]. CNVs are the presence of segments of DNA longer than 1 kb with >90% sequence identity that differ in the number of copies between the genomes of different individuals [5]. It affects more nucleotides per genome than SNP variation [6] and contributes significantly to variation among normal individuals, both in levels of gene expression and in phenotypes of medical relevance [7], [8]. There are many genes and gene families which show copy number differences in population [9]–[11]. One among them is the OR family of genes, having high sequence identity and similar enzymatic functions [1], [12]. OR copy number polymorphisms (CNPs) have been reported in samples covering Africans, Middle Eastern Druze, Southeast Asians, South American Indians, Central American Indians, African-Americans, Yorubans from Nigeria, European descent from Utah, USA, Japanese and Chinese [13], [14]. However, such polymorphisms have not been reported from populations of India or Tibet. We performed a detailed whole-genome copy number scan to investigate the extent of genotypic variations in OR repertoire and to study the genome variability and evolution in populations of India, Tibet along with HapMap samples. Here we report human genotypic variation in functional OR repertoire size, and also the selective pressures acting on CNVs, due to the combinatorial effect of “orthologously obtained” and “paralogously derived” sequences providing the complexity towards CNVs in OR clusters.

## Materials and Methods

For this study, 43 normal members from randomly selected twelve families residing in Karnataka, India, with different age group members ranging from 13–73 years, 270 HapMap samples covering CEU (CEPH collection), CHB (Han Chinese in Beijing, China), JPT (Japanese in Tokyo, Japan) and YRI (Yoruba in Ibadan, Nigeria) populations and 31 Tibetan samples were selected for copy number polymorphism analysis of the OR subgenome. 5 ml EDTA blood was collected from each member of the Indian study group and genomic DNA was extracted using Promega Wizard^®^ Genomic DNA purification kit. The isolated DNA was quantified by Bio-photometer and gel electrophoresis. This research was approved by the University of Mysore Institutional Human Ethics review committee (IHEC). Written informed consent was obtained from all sample donors and the IHEC approved the sample consent procedure. Written informed consent was obtained from parents/guardians in the cases of participants being minors. The 270 individuals sample data from the four populations was obtained from the International HapMap Consortium. The samples for the HapMap come from a total of 270 people: the 30 both-parent-and-adult-child trios from the Yoruba people in Ibadan, Nigeria, 45 unrelated Japanese individuals in Tokyo, 45 unrelated individuals Han Chinese in Beijing, and the 30 both-parent-and-adult-child trios from CEPH. The raw, unprocessed data from Affymetrix Genome Wide SNP 6.0 array for the 31 individuals of Tibet population was obtained from the ArrayExpress Archive at the European Bioinformatics Institute that was submitted by Simonson et al, (2010) to identify regions of the genome that have undergone positive selection in a high-altitude Tibetan population. The deposited datasets were obtained from the ArrayExpress archive with the accession number E-GEOD-21661.

### Genotyping

Genome-wide genotyping was performed using an Affymetrix Genome-wide Human SNP Array 6.0 chip and Affymetrix CytoScan^®^ High-Density (HD) Array having 1.8 million and 2.6 million combined SNP and CNV markers with the median inter- marker distance of 500–600 bases. These chips provide maximum panel power and the highest physical coverage of the genome [15]. Genotyping quality was assessed using Affymetrix Genotyping Console Software. Copy Number Analysis Method offers two types of segmenting methods, univariate and multivariate. These methods are based on the same algorithm, but use different criteria for determining cut-points denoting CNV boundaries.

### BirdSuite (v2)

BirdSuite [16] is a suite originally developed to detect known common CNPs based on prior knowledge, as well as to discover rare CNVs, from Affymetrix SNP 6.0 array data. To do this, it incorporates two main methods; the “Birdsuite” algorithms and the“Canary” [17]. The Birdsuite algorithm uses a Hidden Markov model (HMM) approach to find regions of variable copy number in a sample. For the HMM, the hidden state is the true copy number of the individual's genome and the observed states are the normalized intensity measurements of each array probe. CNV calls from the Canary and Birdsuite algorithms were collated for each sample, and kept as long as they met the following criteria: i) Birdsuite calls with a log10 of odds (LOD) score (Odds Ratio) greater than or equal to 10(corresponding to an approximate False Discovery Rate of ∼5%), ii) Birdsuite calls with copy number states other than 2 were retained; iii) Canary CNP calls with CN states different from the population mode were retained.

### Canary

CNP analysis was performed using the Canary algorithm. Canary was developed by the Broad Institute for making copy number state calls in genomic regions with CNPs. Canary algorithm computes a single intensity summary statistic using a subset of manually selected probes within the CNP region. The intensity summaries are compared in aggregate across all samples to intensity summaries previously observed in training data to assign a copy number state call.

### CNVFinder

CNVFinder developed at the Welcome Trust Sanger Institute uses a dynamic, multiple-threshold based approach to allow robust classification of copy number changes in data of varying qualities. This algorithm makes two main assumptions i) that the majority of data points are normally distributed around a log2 ratio of zero, and ii) that data points falling outside of the centralized log2 ratio distribution are representative of a difference in copy number between test and reference genome.

### Genotyping Console

After processing CEL files and the Birdseed to call genotypes, we used the Genotyping Console (GTC v.3.0.2) to detect CNVs from the Affymetrix 6.0 array for samples that passed initial QCs. The default parameters of >1 Kb size and >5 probes in this algorithm were used.

### Data Analysis

Genome-wide CNV study was carried out using SVS Golden Helix Ver. 7.2 [18] and Affymetrix Genotyping Console software as prescribed in their manuals [19]–[21]. Eigenstrat method was used to avoid possibility of spurious associations resulting from population stratification. Bonferroni correction was employed for multiple testing and the corrected data were then used for CNV testing. Bonferroni methods for population data genotyped on the Affymetrix 6.0 platform was α  = 0.05 thresholds between 1×10^−7^ and 7×10^−8^.

Analyzing the collated data from both BirdSuite and Canary algorithms increased the stringency on those meeting the CNP calls with a log10 of odds score greater than or equal to 10 corresponding to a False Discovery Rate of ∼5%. All SNPs that were called using Birdseed v2algorithm had a Quality Control (QC) call rate of >97% across individuals. All the subjects and members with SNPs that passed SNP QC procedures were entered into the CNV analysis. Filters were set for ID call rates for the overall SNPs to identify IDs with poor quality DNA, if any. The CNV calls were generated using the Canary algorithm. In AGCS, contrast QC has to be >0.4 to be included in the CNV analyses. In this study, contrast QC observed was >2.5 across all samples showing a robust strength. To control for the possibility of spurious or artifact CNVs, we used the EIGENSTRAT approach of Price *et al* (2006) [22]. This method derives the principal components of the correlations among gene variants and corrects for those correlations in the testing. We removed two individuals (1 each individual from India and HapMap-CHB) because they were extreme outliers on one or more significant EIGENSTRAT axes. CNVs were considered validated when there was a reciprocal overlap of 50% or greater with the reference set. Though the Jaccard statistic is sensitive to the number of CNVs called by each algorithm (ideally each two algorithms would detect similar number of CNV calls), the relative values between the different comparisons of algorithms/platform/site are very informative. All the overlap analyses performed have handled losses and gains separately except when otherwise stated, and were conducted hierarchically. The calls from the algorithms that were called in both were not considered; instead, they were collated so that the relative values between the different comparisons of algorithms/platform/site are still very informative.

### HD-CNV

In order to compare and identify CNVs between samples of the same and other population as hotspots and rare, and to also correlate their abeyant effects on a wide variety of biological contexts, HD-CNV [23] (Hotspot Detector for Copy Number Variants), was used to analyze and detect recurrent CNV regions by finding cliques in an interval graph generated from the input. HD-CNV requires CNV calls as an input to detect recurrent regions based on percentage overlap. The output graph generated by the HD-CNV was then visualized using Gephi graph creation software.

### Determining possible recombining regions using MAFFT and BLAST

Both global and local alignment were used to construct the phylogenetic trees. The following two criteria were used to construct the tree, (i) presence of CNVs in OR cluster based on chromosomal location and (ii) divergence of a family of OR genes present on different chromosomal location. Multiple Alignment using Fast Fourier Transform (MAFFT) [24], is a global alignment tool, which is faster, accurate than any other and the trees were constructed using unweighted pair group method with arithmetic mean (UPGMA). Local alignments from BLAST and neighbor-joining methods were used to construct trees. BLAST alignment of sequences was used to construct phylogenetic tree by neighbor joining method.

### Weighted protein interaction network analysis

We used weighted protein network analysis in a first attempt to identify OR gene associated modules and their key constituents. Weighted protein network analysis starts from the level of hundreds of genes, identifies modules of co-expressed proteins and proteins falling under the common pathway and relates these modules to gene ontology information. We made use of tools such as GeneMANIA [25], BIOGRID and CYTOSCAPE [26] developed for network studies to assess the functional consequences of the network topology.

## Results

Whole genome CNP analysis in 344 individuals from the populations covering India, Tibet and HapMap (CEU, CHB, JPT, YRI), homologene analysis, breakpoint analysis, manual curation of the OR sub genome, database scavenging and network studies helped us eventually in identifying 862 OR genes, of which 405(47%) were intact genes and 457(53%) were pseudogenes. During this study significant discrepancies were identified in HORDE, OrDB and HGNC databases while performing data-mining for UOM-HORD. Of the 862 OR genes, 20 genes were not found in HORDE database along with two genes, being wrongly named. Chromosomal locations for 49 OR genes were found to be ambiguous in the HGNC database. Theses discrepancies in the existing OR and other databases paved the way to develop a new OR database. The UOM-HORD which stands for “University of Mysore – Human Olfactory Receptor Database” developed and managed at the University of Mysore is comprehensive and error free.

### UOM-HORD

UOM-HORD is a free online source, which integrates extensive information on human ORs. The UOM-HORD accessible at http://uni-mysore.ac.in/uom-hord/index.php/, was constructed using manual data-mining of the human genome. The human genome was searched for novel and existing ORs and also was cross verified with four other databases HORDE, OrDB, HGNC and NCBI. This data base aims at providing a global overview on the structure, function and evolution of the entire OR gene superfamily. This also furnishes information about OR genomic organization and a comprehensive summary of constantly evolving OR genes in the human genome. The curation of the OR subgenome revealed 405 genes and 457 pseudogenes from 18 families and 292 subfamilies.

### Sub Genome Map of OR clusters underlying CNP

**Figure 1:**
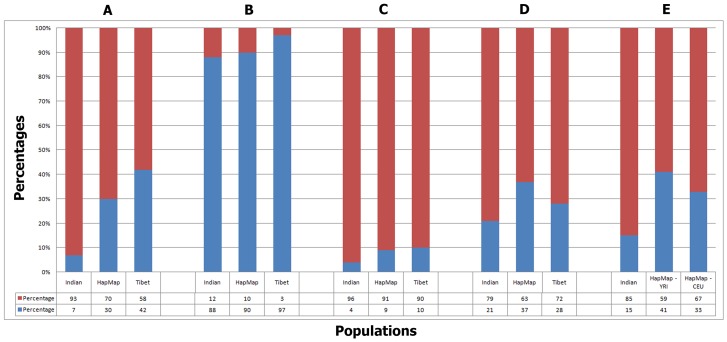
The first, second and third bars in groups A–D represent Indian, HapMap and Tibetan populations. **Group A** denotes percent of subjects with OR genes with CNVs (Red) over subjects without OR genes with CNVs (Blue). 93% of Indian, 70% of HapMap and 58% of Tibetan populations were found to contain CNVs. **Group B** denotes the percent of OR genes with CNPs identified in this study (Red) over percent of OR genes not identified in this study (Blue). 12%, 10% and 3% of the total OR genes were found to contain CNPs in Indian, HapMap and Tibetan populations respectively. **Group** **C** represents the percent of OR intact genes (Red) over pseudogenes (Blue) with CNPs identified in this study. Indian population showed 4% and 96%, HapMap showed 9% and 91% and Tibet showed 10% and 90% of CNPs in intact and pseudogenes respectively. **Group D** represents deletions (Red) over duplications (Blue) identified in this study. Indian, HapMap and Tibetan populations showed 21%, 37% and 28% of deletions and 79%, 63% and 72% of duplications respectively. **Group E** denotes the inherited duplications (Blue 14%) and inherited deletions (Red 1%) whereas stan ds for de novo duplications (58%) and de novo deletions (27%) observed in Indian population whereas, HapMap – YRI trio data denotes 41% and 59% inherited and de novo events the and HapMap – CEU trios shows 33% and 67% inherited and de novo events.

Analyzing the collated data from the arrays from those meeting the CNP calls with a log10 of odds score greater than or equal to 10 (corresponding to a False Discovery Rate of ∼5%) criteria were selected for further investigation. We observed a total of 1527 OR genes from 503 CNV events from all populations. These 1527 OR genes were identified in 81.3% of the entire study group comprising of Indian, Tibetan and HapMap populations. 93% of the Indian population carried OR genes when compared to 58% of the Tibetan population and 70% of the HapMap population (Figure 1). Duplication polymorphism (68%) was significantly high across all populations when compared to deletion polymorphism (32%). These polymorphisms contained ∼16% of the entire OR superfamily of genes, with the Indian population showing the highest degree of gene variability with 12.41% followed by HapMap with 9.86% and the least in Tibet population, with 3.36% (Figure 1). These polymorphisms were found enriched in intact genes than pseudogenes. Some of these polymorphisms in the Indian population were found being inherited, of which 14% and 1% were inherited duplications and deletions respectively (Figure 1), whereas, the remaining 27% and 58% were de novo duplications and deletions, while, the YRI trio data showed 41% and 59% inherited and de novo events followed by 33% and 67% in the CEU trios.

**Figure 2:**
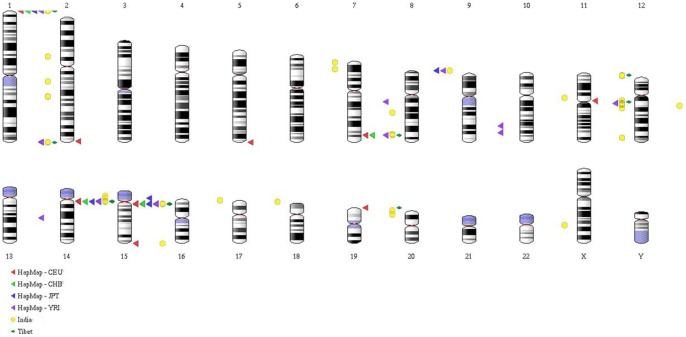
Karyogram indicating the CNPs in different clusters of Indian, Tibetan and Hapmap OR subgenome. Each population is represented by a particular colour. A total of 27 clusters were found from all populations, of which 10 showed overlapping. OR genes are distributed across all chromosomes except 3, 18, 21 and Y. In our study, CNPs were found on all chromosomes containing OR genes except 4, 20, and X.

Figure 2 details the clusters of OR repertoire enriched with copy number identified across all the study populations. The karyogram (Figure 2) shows a total of 27 clusters across all chromosomes from the populations under study. Eleventh chromosome with 6 identified clusters is one of the diverse CNP regions containing both deletion and duplication polymorphisms followed by chromosome 1 with 4 clusters and chromosome 15 with 3 clusters. More number of clusters was identified in the 11^th^ chromosome in the Indian CNP events. Interestingly, 6^th^, 10^th^, 12^th^, 16^th^, 17^th^ and 22^nd^ chromosome clusters were found to be specific only to the Indian CNP event, whereas, chromosomes 2^nd^, 5^th^, 9^th^ and 13^th^ showed cluster specificity only for the HapMap populations and no chromosome specific clusters were identified for the Tibetan population. However, CNPs in six clusters from five chromosomes were conserved across all the study populations whereas; there are no ORs on chromosome 20, and very few on chromosome X(Table 1).

**Table 1:** Showing the distribution of OR clusters containing CNVs in Indian, Hapmap and Tibetan populations.

	Chromosome									
Population	1		7		11		14		15	
India	Cluster	Genes	Cluster	Genes	Cluster	Genes	Cluster	Genes	Cluster	Genes
	1p36.33	4	7q35	6	11q11	7	11q11	10	15q11.2	3
	1q44	14	-	-	11p15.4	6	-	-	-	-
	1q23.1	4	-	-	11q24.1	3	-	-	-	-
	1q23.2	2	-	-	11q12.1	8	-	-	-	-
	-	-	-	-	11q24.2	2	-	-	-	-
**Hapmap CEU**	1p36.33	4	7q35	6	-	-	14q11.2	8	15q11.2	3
**Hapmap CHB**	1p36.33	4	7q35	6	-	-	14q11.2	7	15q11.2	3
**Hapmap YRI**	1q44	20	7q35	5	11q11	14	14q11.2	7	15q11.2	3
**Hapmap JPT**	1p36.33	4	-	-	-	-	14q11.2	6	15q11.2	3
**Tibet**	1p36.33	4	7q35	6	-	-	14q11.2	8	15q11.2	3
	1q44	6	-	-	-	-	-	-	-	-

**Figure 3:**
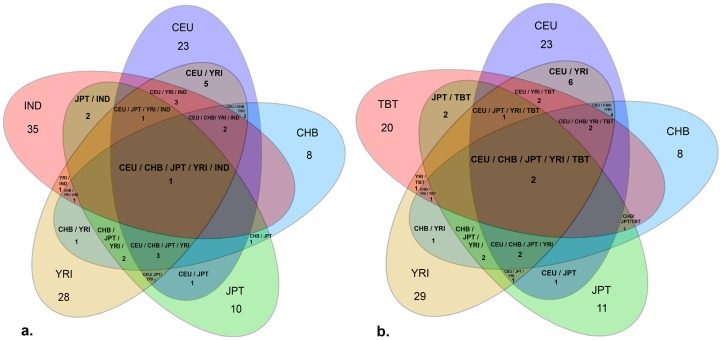
Venn diagrams representing the number of overlapping common Copy Number Polymorphisms (CNPs) found in the six populations studied. (a) represents the CNPs shared between the HapMap populations (CEU, CHB, JPT, YRI) and the Indian (IND) population (b) represents CNPs shared between the HapMap populations and the Tibetan (TBT) population. A single CNP of size 271 kb with the breakpoints 22,317,500–22,588,019 located on chromosome 15 was found in all six populations. Another CNP on the same chromosome with a size of 286 kb, with the breakpoints 22,301,994 bp –22,588,019 bp was shared by five populations and was not found in the Indian population. When both Venn diagrams are taken into consideration, Indian population showed the largest number of exclusive CNP events (35) followed by YRI (29), CEU (23), TBT (20), JPT (11) and CHB (8). The HapMap population showed a maximum of four shared CNPs, Asian populations CHB, JPT, and Tibet share one common CNP while Indian and Tibetan samples share twelve CNPs. European (CEU) and African (YRI) populations share six exclusive CNP events, whereas among the Asian populations, no CNPs were shared between CHB and JPT, as well as CHB and TBT.

Figure 3 further compares the CN events of India and Tibet across the HapMap populations and identified two copy number events, each of 271 kb and 364 kb. 271 kb copy number on chromosome 15q11.2 with breakpoint 22,317,500 bp –22,588,019 bp was conserved across all the study populations, whereas, the 364 kb copy number end point, again on the 15q11.2 chromosome was conserved in HapMap and Tibetan populations only. Indians, Europeans (CEU) and Africans (YRI) show a 3–4 fold heavier load of copy number volume when compared against CHB and JPT copy number volume. Any two randomly chosen populations show a copy number sustenance of 1–6 copy number breakpoints between them. Indian population carry ∼7 OR gene duplications and 2 gene duplications on an average, whereas, members of the Tibetan population show ∼2 gene duplications and ∼1 gene deletion compared to members of the HapMap showing ∼3 gene duplications and ∼ 2 gene deletions. A detailed list of the OR CNV breakpoints common for India, HapMap and Tibet populations has been provided in Table S4.

**Figure 4:**
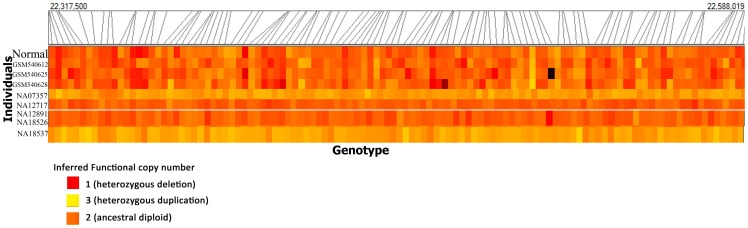
A Heat Map of Log R ratios indicating the quantitative assessments of genotyping examined in a panel of 344 individuals (represented 8) used to determine copy number for the OR region with inferred functional copy number for some of the members under study can be seen here. Each row represents human individuals and each column of the grid summarizes genotype data for the 15q11.2 OR gene cluster comprising of genes *OR4M2, OR4N4* and *OR4N3P*. The slanting lines above the heat emission signal indicate the copy number markers, which have picked the variation. Three Tibetan (GSH540612, GSM540625, GSM540628) samples showing 15q11.2 OR gene cluster deletion and five HapMap (NA07357, NA12717, NA12891, NA18526, NA18537) samples showing both deletions and duplications in the same region.

A Heat Map of Log R Ratios indicating the quantitative assessments of genotyping used to determine copy number for the OR region with inferred functional copy number for some of the members under study can be seen in Figure 4. The slanting lines above the heat emission signal indicates the copy number markers which has picked the variation.

### Hotspot Detection in OR CNPs

**Figure 5:**
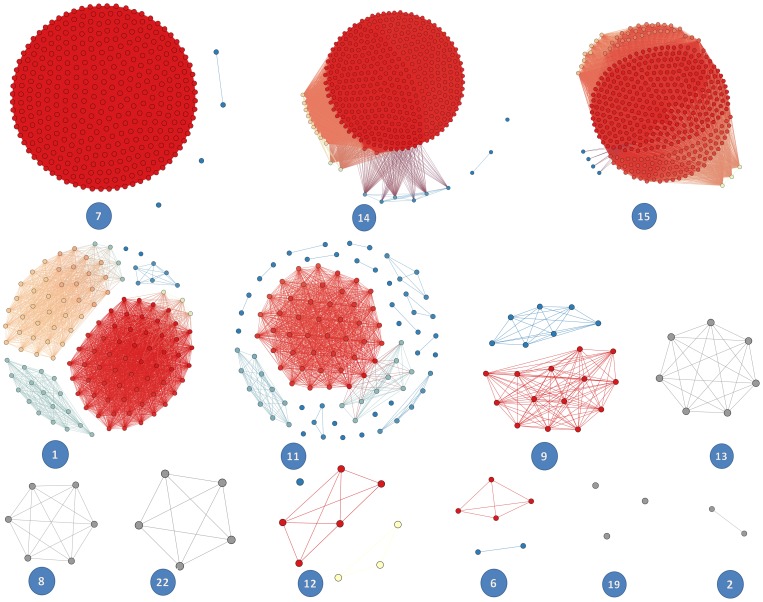
Hot spot detection on OR CNPs was identified using HD-CNV software which generated output files containing overlapping CNV regions, seen as clusters. Red indicates CNV hotspots; Blue indicates rare CNV spots and other colors indicate intermediate CNV events. A total of 1284 hotspots, 77 rare and 137 intermediate OR gene copy number events are distributed across 13 chromosomes.

Hotspot analysis using HD-CNV of the CNPs was used to identify the hotspots and rare CNPs between samples of the same and other population and to also correlate their abeyant effects on a wide variety of biological contexts. About 1527 OR gene calls from this study were used as an input to detect recurrent and rare copy number regions based on percentage overlap. Figure 5 details the detected recurrent CNV regions by finding cliques in an interval graph generated from the input and the graph was visualized using Gephi graph creation software. A total of 1284 Hotspots, 77 rare and 137 intermediate OR gene copy number events are seen in 13 chromosomes (Table S5). Rare events are distinctively limited to only an individual in a population and are not seen in any other individuals in any other population. Varying degrees of Hotspot, Intermediate and Rare ratios were observed in all the chromosomes identified with OR CNVs. Chromosomes 14, 15, 7 and 1 were found to be concentrated with higher ratio of Hotspots. Chromosomes 8, 11, 13, 19 and 22 contain near to equal ratio of hotspots and rare, whereas Chromosome 11 contains highest number of rare events compared to other chromosomes.

### Phylogeny of OR CNPs

**Figure 6:**
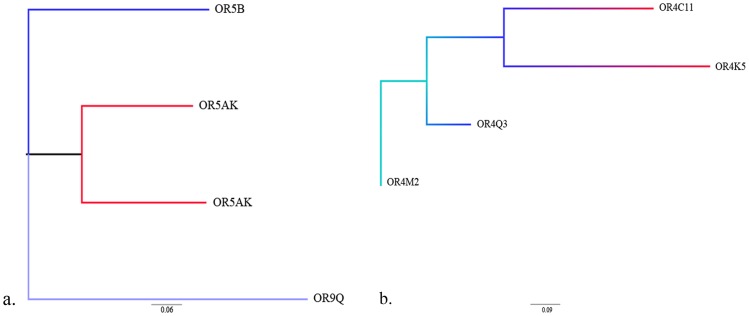
Divergence of CNV across genome. The tree shows the divergence of CNVs present on 11q12 chromosomal location. (b) shows the tree constructed based on the OR4 gene family, which is distributed on different chromosomal locations. Both the trees were constructed by UPGMA method.

Some of the copy number events of size >200 kb present in the 11q11 OR repertoire and also copy number events selected based on the scattered OR4 family genes were aligned discretely as seen in Figure 6 detailing the divergence of some of the OR genes which are under copy number influence. The copy number events aligned based on the family of OR4 were seen scattered across other chromosomes and post this expansion, they seem to diverge more than any. However, when the copy number events were aligned based on the 11q11 chromosomal location, the CNV events of different chromosomes showed more similarity than to the ones which were present on the same chromosome.

### Determining possible recombining regions

In order to annotate the copy number flanking sequences to determine possible recombining regions, −1 kb upstream of the start point and +1 kb downstream of the end point were selected. These sequences were then locally aligned across genomes of other species to determine sequence stretches that are of evolutionary origin and/or derived. Table S1 details the breakpoint analysis of 8 such copy number events varying in size and location, selected based on the recurrent and unique copy number events. For the present study, we used two common CNV events present in more than three populations and a rare/unique CNV event.

The 271 kb CNV is located on chromosome 15q11.2 with the break points 22317500 bp-22588019 bp with 113 markers comprising of the genes *OR4M2, OR4N4* and *OR4N3P*. It is a common CNV present in all 6 populations – HapMap (YRI/JPT/CEU/CHB), India and Tibet, and thus was taken for the analysis. A total of two common CNVs (including the above described 271 kb CNV) present in 5 or more populations and the other, a 318 kb event on 14q11.2 consisting of *OR11H2, OR4Q3, OR4M1, OR4N2, OR4K2, OR4K5,* and *OR4K1* were taken to begin the analysis. In addition to these breakpoint CNVs, three other extended endpoints for the same common start point of 22,317,500 bp on 15q11.2 and for one rare 410 kb event with breakpoints 19802529 bp –20212323 bp on 14q11.2 were also included in the analysis. UCSC Genome Browser Build hg18/NCBI Map Viewer Build 36.3 was used to obtain information on the genomic segments involved in the CN detection and for the corresponding list of genes (Table 2).

**Table 2:** Details of CNV breakpoints considered to determine the possible recombining regions.

Start Point	End Point	Size	Location	Start Marker	End Marker	Total Markers	Genes
**Common**							
20105479	20423360	318	14q11.2	CN_657383	CN_105694	114	OR11H2, OR4Q3, OR4M1, OR4N2, OR4K2, OR4K5, OR4K1
22317500	22588019	271	15q11.2	CN_680698	SNP_A-2133472	113	OR4M2, OR4N4, OR4N3P
**Common start point - Alternate end points**							
22317500	22681064	364	15q11.2	CN_680698	CN_680783	115	OR4M2, OR4N4, OR4N3P
22317500	22474268	157	15q11.2	CN_680698	CN_680748	64	OR4M2, OR4N4, OR4N3P
22317500	22494283	177	15q11.2	CN_680698	CN_680756	74	OR4M2, OR4N4, OR4N3P
**Rare**							
19802529	20212323	410	14q11.2	CN_657370	CN_657395	18	OR11H2

The most common copy number events of chromosome 15q11.2 described above and found in more than 3 populations were used to begin the analyses. The maximum threshold percent overlap for the local alignment across species and within the genome was set to 95%. The upstream flanking sequences of the 271 kb event, the most common event with start point 22,317,500 shows a total of 12 identity hits with 97% to 100% overlap, with 8 hits from regions within the genome and 4 hits with closely related species. Though, most identity hits were on the same 15^th^ chromosome, chromosome 14 with about 97% identity was found to be the nearest in origin. However, the other 4 identity hits were seen across other closely related species *Rhesus Macaque* (chromosome unknown) with 97%, *Pan troglodytes* (Chromosome 22) with 98%, *Pongoabelli* (both chromosome 14 and 12) with 96% identities.

The downstream flanking sequences of the endpoint (22,588,019) revealed 7 identity hits, most being on the same 15^th^ chromosome, but chromosome 2 with about 94% identity was found to be the nearest in origin. There were no ancestrally derived sequence hits observed for any of the closely related species. We continued the analyses to include other identified endpoints in the same vicinity for the common start point described above (22,317,500), for the 22,681,064 bp –22,682,064 bp region, and found 14 identity hits extending from 96% –100%. Chromosome 13 of *Homo sapiens* and chromosome 22 of *Pongoabelli* showed 100% sequence identity, whereas, chromosome 8 clone RP11-10H3 showed 96% sequence identity along with varying degrees of identity for chromosome 15. For the 22,494,283 bp –22,495,283 bp region, we found 5 identity hits extending from 95% –100%. Chromosome 12 of *Homo sapiens* showed 95% sequence identity, whereas, varying degrees of identity for chromosome 15 on several clones were observed. For the 22,474,268 bp –22,475,268bp region, we found 9 identity hits extending from 96% –100%. Chromosome 14 of *Pan troglodytes* showed 97% sequence identity, whereas chromosome 17 clone CH17-224D4 and chromosome 14 clone CH17-262H11 showed 96% sequence identity along with varying degrees of identity for chromosome 15.

**Figure 7:**
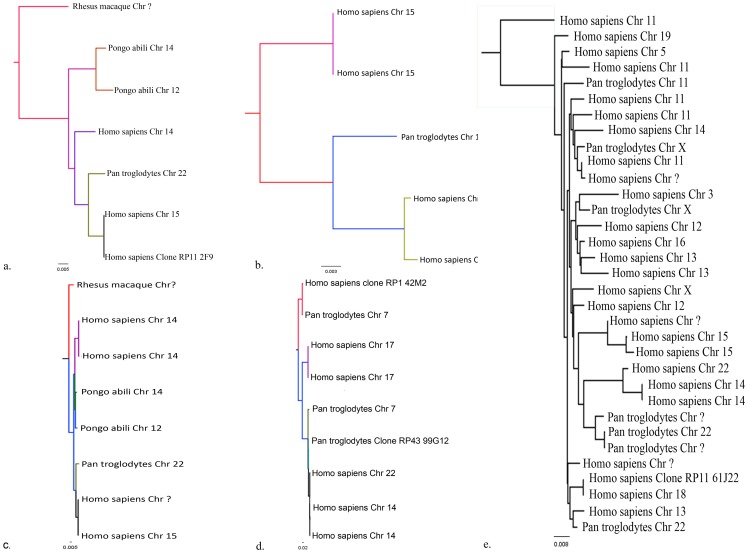
Phylogenetic tree of the flanking recombining upstream and downstream sequence breakpoints of CNV regions – pattern of dispersal of breakpoint across taxa and within the genome of an organism. a–d show the trees for the −1 kb upstream and downstream flanking sequences of the most common CNV event found in all the populations under the study. Figure 7e shows the tree for the −1 kb upstream flanking sequences of rare CNV event found in a population under study. These flanking sequences contain the recombining regions, whose relationship with other orthologous and paralogous sequences can be seen to determine the origin of these sequences and probable other recombining regions. The −1 kb upstream and downstream flanking sequences of the most common CNV events (22,316,500 bp –22,317,500 bp in 15q11.2), (22,474,268 bp –22,475,268 bp in 15q11.2), (22,681,064 bp –22,682,064 bp in 15q11.2), (20,104,479 bp –20,105,479 bp in 14q11.2), (19,801,529–19,802,529 in 14q11.2) found in all the populations under the study were chosen to construct the trees. Figure 7e shows the tree for the −1 kb upstream flanking sequences of rare CNV event found in a population under study. These flanking sequences contain the recombining regions, whose relationship with other orthologous and paralogous sequences can be seen to determine the origin of these sequences and probable other recombining regions.

Another common CNV event of 318 kb with breakpoints (20,105,479 bp –20,423,660 bp) from chromosome 14q11.2 was also included in the analyses, the 20,105,479bp –20,104,479bp upstream flanking sequence of the copy number start point showed 10 identity hits, an unknown chromosome with RP43-26N14 clone showed 98% and chromosome 7 of *Pan troglodytes* showed 95% along with 95% –99% identity for *Homo sapiens* chromosomes 22, 14 and 7. The downstream flanking sequences however showed hits only to regions within the same chromosome. A 410kb rare copy number event with breakpoints 19,802,529 bp –20,212,323 bp, again on chromosome 14q11.2 with upstream flanking region of 19,802,529bp start point showed 81 identity hits on human chromosomes and 14 hits on genomes of other species. These 81 hits were found across chromosomes 1, 2, 3, 4, 5, 11, 12, 13, 14, 15, 16, 18, 19, 20, 22 and X chromosomes and the sequence identity ranged from 95%−100%. Similarly, chromosomes 2, 7, 14, 22, X and some unknown chromosomes of *Pan troglodytes* showed 96% identity hits. The downstream flanking sequences however showed hits to chromosome 15 (95%) of *Homo sapiens* and chromosomes 12 and 14 (97%) of *Pongoabelli*. These flanking sequences contain the recombining regions, whose relationship with other orthologous and paralogous sequences can be seen to determine the origin of these sequences and probable other recombining regions (Figure 7). Phylogenetic trees of the breakpoint regions are mimicking the expansion of CNV across taxa. For the present study, we used two common CNV events present in more than three populations and a rare/unique CNV event (Figure 7).

### Protein Interaction network of Human Olfactory receptor genes

**Figure 8:**
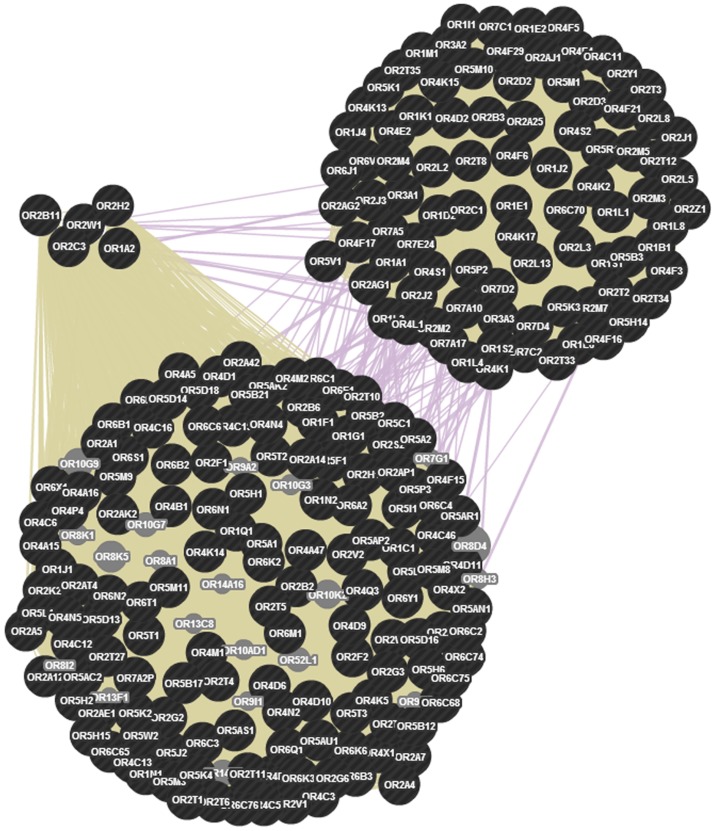
Network of genes involved in olfactory perception with hub genes distributed in three clusters. The network shows 135 genes (blue nodes), their co-expression (grey lines) and shared protein domains (green lines) that were identified in our study. About 50 genes are co-expressed at the transcript level; however they are inhibited by negative feedback supporting the one neuron–one receptor hypothesis. All OR genes show shared protein domains. The network generated has a clustering coefficient of 0.9, network density is 0.431 and network heterogeneity is 0.266. Each gene has an average of 57.8 neighbors.

Figure 8 describes the network topology of the 151 OR genes, with co-expression and shared protein domain features divided into 3 clusters. Eleven of the OR genes in this network are seen co-expressing with only a few of the other OR genes belonging to both same and different families. Figure S1 details the network statistics with respect to network neighbors, shared neighbors and stress centrality of the OR network. This network has 135 nodes with a network density of 0.431, has about 70 multi-edge node pairs, and has ∼57 average numbers of neighbors, with a clustering coefficient of 1.

OR genes identified in the CNV events of this study were used to construct OR protein network in order to determine the physical interactions of the OR protein receptors. No physical interaction was observed for the OR proteins that were used from the study. However, some of the OR proteins among the ones that were used to begin the network construction were found to be co-expressed. The co-expression pattern is on par with the one receptor one neuron hypothesis proposed by Malnic *et* *al* (1999) [27] wherein a single gene among the expressed OR primary transcripts, is randomly chosen to be transformed into a OR G-protein coupled receptor and regulates the other expressed OR transcripts through a negative feedback mechanism. About 50 OR genes from a set of 135 genes were found to be co-expressing at the mRNA level, but due to inhibition, they fail to co-express at the protein level resulting in the formation of a single receptor for every neuron, as supported by the generated OR protein interaction network. CNV burden on OR genes may possibly alter the copy number states of OR genes which in a way affects the co-expression levels of the transcripts resulting in the increased or decreased negative feedback mechanism during the determination of OR transcript to OR receptor pathway. Therefore the protein interaction network of OR genes is established.

A complete list of the OR genes and the OR CNV events with detailed coordinates, its CNV type, location, size and markers identified in India, HapMap and Tibet populations has been provided as a Table S3.

## Discussion

Multigene families comprise of genes that are identical or having similar sequence and the similarity can be either for the entire sequence or partial, limited to specific domains. These multigene families are seen scattered across chromosomes or localized at one place [10]. There are >10 such multigene families in human genome. The genes for alpha and beta chains of the mammalian hemoglobin molecule are coded by multigene families on chromosomes 16 and 11. Multigene families of actins, immunoglobulins, interferons, tubulins, hemoglobins and histones are seen scattered and conserved [11]. The most prominent in the human genome, the rRNA genes, alone has 2000 genes for 5S rRNA. The most over represented category was the family consisting of alpha-amylase multigene family (AMY1 and AMY2) located on chromosome 1. The second most frequent being the uridine glucuronosyltransferase (UGT) gene family divided into two subfamilies, UGT1 and UGT2 located on 2q37 and 4q13 respectively. Similar to UGT1, the region for the protocadherin beta (PCDHB) gene family encodes 16 different proteins with variable N-termini. OR gene family among these multigene families is the largest, occurring in both human and lower primates [1]. These multigene families are generated by continuous genomic rearrangements caused by duplications, deletions, and inversions in the genome [28].

The human genome contains ∼100 OR clusters, that has arisen via numerous tandem duplications, as well as less frequent interchromosomal duplications, that seeded new clusters and harbors between 1–105 genes [29]. Some human ORs have multiplied to relatively high copy number as part of a recent burst of segmental duplications in the human genome [29]. OR gene loci are known to be one of the most genetically diverse regions in the human genome. In 2003, 388 intact OR genes and 414 OR pseudogenes were reported from the human genome sequence [30], however, the numbers improved to 396 intact and 425 pseudogenes in 2010 due to the release of the updated human genome build [31]. But recent studies accounted OR repertoire into 413 intact genes and 244 segregating pseudogenes [32].We have carried out high resolution CNV analysis of OR repertoires in 344 individuals from 3 sample groups, and eventually identified the OR intact and pseudogenes to be 862, and we have also identified OR containing CNVs which were not earlier reported.

### Validations

Firstly, 51440 CNVs including HapMap samples were detected by Zhang et al (2011) [33] from the HapMap samples, 90 HapMap – CEU samples were validated by Conrad et al (2011) [34]. Both of them have used algorithms such as Birdsuite, Partek, PennCNV-Affy and HelixTree. These both studies have been considered for CNV calling as gold standard. Similarly, we have also analysed HapMap CNV events using BirdSuite, Canary and CNVFinder. In the above algorithms, the recovery rate also increased with the number of probes spanned by the CNV. Secondly, comparison was made with the 1695 structural variations reported by Kidd *et al* (2008) [35] from 8 human genomes which revealed an impressive degree of overlapping for our data even for the larger CNVs.

We used 893 CNVs which was used by Korn *et al*. (2008) [36] as a reference to compare the recovery rates of Birdsuite, Canary and CNVFinder for the 1042 HapMap CNV events detected in our study. Birdsuite recovered CNVs spanning >5 markers at a rate (81.5%) comparable to that of Korn *et al* (2008) [36] (92.4%) when using their criteria. We have also compared 1042 HapMap CNV events with the 893 CNVs reported by Korn *et al* (2008) [36] where the Birdsuite algorithm recovered more than 90% of the CNVs. However, the highest recovery rate for detection of CNVs with >5 markers was significant by Birdsuite. We further calculated the average recovery rate of CNVs with different frequency spanned by >5 makers. The array consists of ∼22000 labeled reference genes. Though some OR genes were not labeled in the array, they were identified when the CNV breakpoints were checked in the NCBI genome maps. Both OR intact and pseudogenes were also verified in the NCBI and HORDE databases. This indicates that both OR intact genes and pseudogenes are equally represented in the array. The complete list of OR genes present in the array are provided in Table S2.

While performing several layers of analysis, we identified significant discrepancies in the two OR databases, HORDE and OrDB leading to the creation of the new database “UOM-HORD”. UOM-HORD houses the gene information, symbol status, and nucleotide information of all the identified and annotated OR genes. This database aims at providing OR subgenome structure, function and organization of constantly evolving OR genes from 18 families and 292 subfamilies.

### Subgenome map of OR clusters

Our study focused CNPs in regions of both single gene loci and OR clusters across all chromosomes using Affymetrix Genome-Wide Human SNP Array 6.0 chip and Affymterix CytoScan^®^ High-Density (HD) Array having 1.8 million and 2.6 million combined SNP and CNV markers with the median inter- marker distance of 500 bps in the genome in contrast to earlier low-resolution studies (75–100 kb to 1 kb) [37], [38]. Comparing the coordinates of the CNPs and OR gene loci/clusters, we found that a high volume of the intact genes were found enriched in CNP regions compared to pseudogenes. Indian, Tibetan and HapMap samples showed varying percent of copy number occurrence, with India being the highest at 93%, followed by HapMap and Tibet. This study group contained overlapping OR loci, and covered ∼16% of the total 862 OR loci, compared to ∼15–20% OR genes found to be affected by CNVs as reported by Waszak et al [39]. Duplication polymorphism were significantly enriched across all populations compared to deletion polymorphism and HapMap contained the lowest events. Indian trio data revealed 15% of the copy number being inherited compared to 85% being *de novo* events which can be attributed to the heterogeneity of the Indian genome, compared to 30% and 16.3% being inherited and de novo in YRI and 25% and 30% being inherited and de novo in CEU subgroup.

Go and Nimura (2008) [40] suggested that the pace of evolution of OR genes is similar between humans and chimpanzees, but the OR gene repertoires were found to be relatively different between them. This difference might be responsible for the species-specific ability of odor perception. Primates are less dependent than mouse and dog on an olfaction, which might have been due to a measured gene loss process along the lineage. Similar OR repertoire losses have been reported in other mammals [41], [42], [32]. Malnic et al., (2004) [3] constructed a phylogenetic tree in which they showed sequence relationships among the 339 human ORs, 23 rodent ORs with known ligands, and 28 ORs identified in fish. Gilad et al., (2004) [43] sequenced 100 genes from 18 nonhuman primates. In higher apes, the gene loss has extremely accelerated in humans. Such loss in the functional OR repertoire in humans is an ongoing evolutionary process, as established by the past identification of OR genes that segregate between intact and pseudogene forms and by newer surveys showing an enrichment of loss-of-function OR alleles^32^ (Olender et al., 2012) [32]. The CNV events and segmental duplications are some of the reasons why OR genes/clusters are scattered across the chromosome from lower to higher order species. The recent developments in CNV detection post the genomic era have enabled one to detect the extensive distribution of ORs through CNVs.

### Hotspot Detection in OR CNPs

In order to identify the hotspots and rare CNPs between samples of the same and other population, HD-CNV (Hotspot Detector for CNVs) program was used on 1527 copy number events revealing hotpot, rare and intermediate regions. HD-CNV showed chromosome 7 having extensive hotspot events. Except for a few unique CNV events, almost all events overlap with single (or almost) other event. These events are very commonly recurring events that have similar boundaries, not much distinction can be observed in the groups. Chromosome 15 shows a large group, all of which share some CNPs and then a few that do not share among themselves but share with a CNV that is common to almost all. Intermediate subgroups in chromosomes 1 and 11 are seen to be interconnected to each other, but not directly connected. Intermediate copy number events are those which are neither hotspots nor rare, are bound to be influenced either ways. There are few individual events that are not shared by a single unique event. In chromosome 14, a large group that all share together, and then a few events that do not have all of those events but do overlap with at least one in the group, gets joined.

Hot-Spot Detector imports pre-formatted CSV files containing detected CNV events in the study. CNV events are treated as nodes in an interval graph and are used to represent regions (intervals) on a real line, and edges are added where intervals overlap as postulated by Lekkerkerker *et al.*, (1962) [44]. Based on this, Butler *et al* (2012) [23], modified and added edges between nodes that share the base pair overlap required to consider two CNV events part of a merged region (default 40%) and the overlap required for a family (region with highly similar CNV events, default 99%). Merged regions, therefore, contain a collection of CNV events where each overlaps all others in the merged region by the minimum overlap specified, and indicate the genomic location where those groups of overlapping CNVs are found [23].

Hotspot analysis elucidates the fragility of the OR subgenome which contains recurrent CNV regions bearing large concentration of repeats in the flanking sequences. Some intermediate subgroups are seen to be shared with both hotspot clusters and rare clusters indicating that the subgroup is under selective pressure and have equal chances of being converted into either clusters. Intermediate subgroups in the chromosomes are at a dynamic state and tend to shift either towards hotspots or rare over a continuous evolutionary CNV burden. However, this state seems to be inert in nature currently, with little or no scope for state conversion but may tend to change over a period of evolutionary CNV burden. Hence the intermediate subgroups are regarded to be under selective pressure.

Phylogenetic trees were constructed aligning >200 kb CNPs from several chromosomal locations identified in this study to address questions relating to the evolution of the OR repertoire and to detect the role of a neutral evolution for the OR copy number variation as proposed by Nozawa et al [32]. Sequences were chosen based on two criteria, one was using sequences from a defined cluster of a chromosomal location, and other being, based on the CNP sequences of the same OR family of genes, but scattered across several locations. Chromosomal location 11q12 bearing five genes from OR5 family along with the use of four CNP sequences occurring in OR4 family of genes were used distinctly to determine the divergence of some OR genes/sequences under copy number influence. Phylogenetic trees gave interestingly contrasting states, the tree with the scattered OR4 genes seemed to diverge more than the tree constructed on the CNP from a chromosomal cluster (Figure 6). The CNV events showing less or no divergence between different chromosomal locations might implicate recent expansion into the new location. CNV formation bias was observed in the OR repertoires as OR intact genes were significantly affected by CNVs than OR pseudogenes with stark contrast to other studies [38] which indicates the evolutionary constraints acting on OR intact genes than on pseudogenes indicating an intentional contribution of formation bias and selection.

### Role of Orthologous – Paralogous sequences in OR gene clusters

Having established the absence of neutral theory and intentional contribution of formation bias and selection on CNVs towards OR intact genes, we further tested the recurrent and unique CNP events for their flanking recombining sequences to decipher the complexity involved in the insistent CNVs in the OR repertoires. In order to annotate the copy number flanking sequences and to determine possible recombining regions, −1 kb upstream of the start point and +1 kb downstream of the end point were selected. These sequences were then locally aligned across genomes of other species to determine sequence stretches that are of evolutionary origin and/or derived. It is likely that the duplication and deletion processes are altering the OR family by homologous as well as non-homologous unequal recombination between the OR regions. The OR clusters have arisen via numerous tandem duplications, as well as less frequent interchromosomal duplications, that seeded new clusters [29]. These human ORs have multiplied to relatively high copy number as part of a recent burst of segmental duplications (SDs) in the human genome. Numerous studies have tried to identify the reasons behind such continuously occurring CNVs, many have failed to unravel the complexities of the CNV occurrence and its burden on the OR gene regions [10].

In order to unravel the complexities surrounding the recurring CNV events in the OR repertoire, we used a) the phylogeny of OR CNPs to determine if the recombining regions of a CNV are ancestral or derived sequence segments and b) to determine the possible recombination regions both intra- and inter-chromosomally for some of the Hotspot and rare CNV start and end points. The conserved flanking sequences of CNVs of ORs from the closely related species were compared, of which 271 kb and 364 kb CNVs were found to be the two most commonly shared CNV events across all populations. These two CNV events were used to find the ancestry of the sequences. The analysis was begun by obtaining −1 kb upstream flanking sequence of the start point and +1 kb downstream flanking sequence of the end points of 271 kb, 318 kb hotspots and 410 kb rare CNV. These sequences were then locally aligned across genomes of closely related species *Pongo abelli*, *Pan troglodytes* to determine sequence stretches that are of evolutionary origin and/or derived. The threshold percent of overlap for the local alignment across species and within the genome was set between 99% –95% sequence identity. Sequence identity hits were observed for the closely related species as well as for the other chromosomes in the *Homo sapiens* genome. Based on the closest sequence identity hits for these CNV events, the flanking sequence stretches were regarded to be either evolutionary or derived. With the above information, we can consider the flanking sequence of 271 kb CNV as ‘orthologous’ whereas another CNV of 177 kb with the same start point but different end point from the 271 kb can be considered as ‘derived’. Based on the degree of sequence similarity both across chromosomes and across closely related species, the closest hit in sequence identity was regarded to be a hotspot region/probable region for recombination. In this way, the recombination regions were predicted based on the flanking sequence identity hits across the chromosomes and within the genome. The flanking sequence stretches were also analyzed for the presence of genomic repeat sequences and CpG islands while determining the recombination regions. The genomic repeat sequences were few and we did not find the interference of the repeat sequence elements for recombination.

Though studies have shown that ORs with a closely related paralog are significantly more likely to be affected by CNVs than ORs lacking a closely related paralog, however, we see that it is the sequences flanking the OR containing genes rather than the OR genes themselves, which is probably the reason why the opposite was observed in other studies. We note that, these upstream and downstream flanking sequences show identity hits ranging 95% –100% in regions of other chromosomes within the genome and also across other species such as *Pan troglodytes*, *Pan abelli* and *Rhesus Macaque*. Interestingly, we also found hits to non-ancestrally derived sequences with other chromosomes within the genome (Table S1). These derived sequences do not have any ancestral origin for any closely related species but show stark identities to regions of other chromosomes within the genome indicating the role of ancestral orthologous and evolutionarily new derived paralagous sequences. It is this combinatorial effect of both “orthologous obtained from closely related species” and “paralogous derived sequences” which are providing the complexity to the continuously occurring OR CNVs. The mechanism involved in generation of OR copy-number is long thought to be because of the ‘Non Homologous End Joining’ and ‘Non Allelic Homologous Recombination’ [38]. We believe these recombination events are due to the extensive sequence identities of the flanking sequences with several regions (1 to 50) across other chromosomes. These regions are the probable recombining sequence stretches which has transported OR loci/clusters to other regions in the genome.

### OR Protein Network follows one gene – one neuron hypothesis

The OR protein interaction network was created using 140 identified OR genes in our study, which showed the interactions between different OR family genes. The genes formed three clusters, all of which had shared protein domains and some being co-expressed. The network showed no physical interaction between the genes and no co-localization. The co-expression pattern of the genes throw a light on the transcriptional regulatory mechanism [45]–[46] wherein a negative feedback is seen and the OR protein formed inhibits the translation of other OR transcripts. The co-expression pattern is on par with the one receptor-one neuron hypothesis wherein a single gene expressed randomly in every neuron gives rise to a receptor. About 11 genes were found to be co-expressing at the mRNA level, but due to inhibition, they fail to co-express at the protein level resulting in the formation of a single receptor for every neuron, as supported by the generated OR protein interaction network.

This is a maiden report of olfactory copy number study in a total of 344 individuals from 3 sample groups of India, Tibet and HapMap revealing the presence of CNPs in olfactory receptor gene regions. These OR CNVs were studied to unravel the complexities involved in the recurrent CNV burden regions. There was a combinatorial effect of both “orthologously obtained sequences from closely related species” and “paralogous derived sequences” which are providing the complexity to the continuously occurring OR CNVs.

## Supporting Information

Figure S1 OR protein network containing 135 OR genes were analysed using NetworkAnalyzer considering it as an un-directed network. NetworkAnalyzer was also used to perform topological analysis containing both undirected and directed edges. (a) Displays the node degree of nodes (genes) having 80 edges linked to each, where in-degree distribution and out-degree distribution can be observed to distinguish between random and scale-free network OR topologies. (b) Displays the number of neighbors connectivity of node and is the number of its neighbors with respect to the average clustering coefficient. The neighborhood connectivity of a node n = 80 is the average connectivity of all neighbors of the entire OR gene network. (c) displays the number of neighbors connectivity of node and is the number of its neighbors with respect to the topological coefficient. The numerical attribute is clustered at 0.56 coefficient of n = 80 which should have a minimum of 2 neighbors to have a topological coefficient of zero. (d) shows the frequency of the nodes (OR genes) with respect to path length distribution. 4 path lengths across significantly varying frequency were observed for the edges between the OR gene nodes. (e) Number of shared neighbors for a given OR gene node can be seen here, with the large cluster containing ∼65 neighbors with a higher frequency followed by ∼1 neighbor with a much lower frequency. (f) Shows the n = 80 neighbors clustered at the average neighborhood connectivity of 60–70 neighbors each. (g) displays the betweenness centrality of all the neighbors in this network, the betweenness centrality value for each nodes is normalized by dividing by the number of node pairs shown along with the Fit of Power Law. (h) shows the closeness centrality of OR genes plotted against the number of OR gene neighbors with the reciprocal of the average shortest path length, the closeness centrality of each OR gene node is a number between 0 and 1 shown along with the Fitting line. (i) Displays the stress centrality of the OR gene multiple edge network, where each OR gene node is the number of shortest paths passing through each of the other nodes. OR gene nodes are shown to have high stress as it is traversed by a high number of shortest paths. The stress centrality is seen distributed at 5 regions, OR gene nodes are seen at 1, 1E2, 1E3 and 1E4 containing 60 OR gene nodes.
(TIF)

Table S1 Describes the −1 kb/+1 kb upstream and downstream sequences of the copy number breakpoint, 22,317,500 bp –22,474,268 bp and its homology within and across the genome of closely related ancestral species. These breakpoints help to understand the complexity of two distinct features of evolutionarily conserved and derived sequences of the genome. The columns following that denotes the upstream sequences (−1 kb) of breakpoint start region, followed by columns containing the percent of similarity. The other half of the Table shows regions of the upstream breakpoint that's has the highest identity hits in ancestral species and their % similarity. Ninth column shows the downstream region of the breakpoint end (+1 kb). Tenth and eleventh column contains other regions of breakpoint in humans and their % similarity. Twelfth and thirteenth column shows other sequence regions of downstream breakpoint region that have high sequence identity in ancestral species along with their % similarity.
(DOC)

Table S2 Complete list of Olfactory Receptor genes and pseudogenes in humans along with the list of Intact genes  = 405–46.98%, Pseudogenes  = 457–53.01%, and Total OR genes  = 862.
(XLSX)

Table S3 List of CNV events, cytoband positions, size, count, and markers identified across HapMap, Tibet and Indian population.
(XLSX)

Table S4 Representation of the number of overlapping common Copy Number Polymorphisms (CNPs) found in the five populations studied including four from HapMap and Tibetan populations. CEU (CEPH collection), CHB (Han Chinese in Beijing, China), JPT (Japanese in Tokyo, Japan) and YRI (Yoruba in Ibadan, Nigeria), TBT (Tibetan). Y represents ‘Yes’ indicating the presence of that CNV in that particular population.
(DOC)

Table S5 Hotspot analysis using HD-CNV of the CNPs was used to identify the hotspots in chromosomes of various populations.
(DOC)

## References

[1] Hasin-Brumshtein Y, Lancet D, Olender T (2009) Human olfaction: from genomic variation to phenotypic diversity. Trends Genet 25(4): 178–184. 19303166 10.1016/j.tig.2009.02.002

[2] Fuchs T, Glusman G, Horn-Saban S, Lancet D, Pilpel Y (2001) The human olfactory subgenome: from sequence to structure and evolution. Hum Genet 108(1): 1–13. 11214901 10.1007/s004390000436

[3] Malnic B, Godfrey PA, Buck LB (2004) The human olfactory receptor gene family. Proc Natl Acad Sci U S A 101(8): 2584–2589. 14983052 10.1073/pnas.0307882100 PMC356993

[4] Kambere MB, Lane RP (2007) Co-regulation of a large and rapidly evolving repertoire of odorant receptor genes. BMC Neurosci 8 Suppl 3S2. 17903278 10.1186/1471-2202-8-S3-S2 PMC1995454

[5] Freeman JL, Perry GH, Feuk L, Redon R, McCarroll SA, et al (2006) Copy number variation: New insights in genome diversity. Genome Res 16: 949–961. 16809666 10.1101/gr.3677206

[6] Redon R, Ishikawa S, Fitch KR, Feuk L, Perry GH, et al.. (2006) Global variation in copy number in the human genome. Nature 444, 444–454. 10.1038/nature05329 PMC2669898 17122850

[7] Aitman TJ, Dong R, Vyse TJ, Norsworthy PJ, Johnson MD, et al (2006) Copy number polymorphism in Fcgr3 predisposes to glomerulonephritis in rats and humans. Nature 439: 851–855. 16482158 10.1038/nature04489

[8] Gonzalez E, Kulkarni H, Bolivar H, Mangano A, Sanchez R, et al (2005) The Influence of CCL31gene-containing segmental duplications on HIV-1/AIDS susceptibility. Science 307: 1434–1440. 15637236 10.1126/science.1101160

[9] Sudmant PH, Kitzman JO, Antonacci F, Alkan C, Malig M, et al (2010) Diversity of Human Copy Number Variation and Multicopy Genes Science. 330(6004): 641–646. 10.1126/science.1197005 PMC3020103 21030649

[10] Niimura Y, Yoshihito G (2012) Olfactory Receptor Multigene Family in Vertebrates: From the Viewpoint of Evolutionary Genomics. Curr Genomics 13: 103–114. 23024602 10.2174/138920212799860706 PMC3308321

[11] Kim HL, Iwase M, Igawa T, Nishioka T, Kaneko S, et al.. (2012) Genomic Structure and Evolution of Multigene Families: “Flowers” on the Human Genome. Int J Evol Bio doi:10.1155/2012/917678. 10.1155/2012/917678 PMC3388347 22779033

[12] Hoppe R, Frank H, Breer H, Strotmann J (2003) The Clustered Olfactory Receptor Gene Family 262: Genomic Organization, Promotor Elements, and Interacting Transcription Factors. Genome Res 13(12): 2674–2685. 14656972 10.1101/gr.1372203 PMC403809

[13] The International HapMap Consortium (2003) The International HapMap Project. Nature 426: 789–796. 14685227 10.1038/nature02168

[14] Simonson TS, Yang Y, Huff CD, Yun H, Qin G, et al (2010) Genetic evidence for high-altitude adaptation in Tibet. Science 329: 72–75. 20466884 10.1126/science.1189406

[15] Affymetrix Inc. (2009) Data Sheet: Genome Wide Human SNP Array 6.0.

[16] BirdsuiteAlgorithm http://www.broad.mit.edu/mpg/birdsuite/birdseed.html (Accessed September 15th 2010).

[17] White Paper: Affymetrix^®^Canary Algorithm Version 1.0.: 1–7 (2008)

[18] Bozeman MT: Golden Helix, Inc. SNP & Variation Suite (Version 7.x) [Software]. Available from http://www.goldenhelix.com (Accessed Oct 1 2012).

[19] Affymetrix Inc. (2005) Technical Note: Guide to Probe Logarithmic Intensity Error (PLIER) Estimation.

[20] Affymetrix Inc. (2007) White Paper: BRLMM-P: A Genotype Calling Method for the SNP Array 5.0.

[21] Affymetrix Inc. (2008) User manual: Genotyping Console™ Software 2.1.

[22] Price AL, Patterson NJ, Plenge RM, Weinblatt ME, Shadick NA, et al (2006) Principal components analysis corrects for stratification in genome-wide association studies. Nat Genet 38 (8): 904–9. 10.1038/ng1847 16862161

[23] Butler J, Locke M.E, Hill KA, Daley M (2012) HD-CNV: Hotspot Detector for Copy Number Variants. Bioinformatics. 10.1093/bioinformatics/bts650 23129301

[24] Katoh K, Kuma K, Toh H, Myata T (2005) MAFFT version 5: improvement in accuracy of multiple sequence alignment. Nucleic Acids Res 33: 511–518. 15661851 10.1093/nar/gki198 PMC548345

[25] Warde-Farley D, Donaldson SL, Comes O, Zuberi K, Badrawi R, et al (2010) The GeneMANIA prediction server: biological network integration for gene prioritization and predicting gene function. Nucleic Acids Res 1: 38–45. 10.1093/nar/gkq537 PMC2896186 20576703

[26] Shannon P, Markiel A, Ozier O, Baliga NS, Wang JT, et al (2003) Cytoscape: a software environment for integrated models of biomolecular interaction networks. Genome Res 13: 2498–2504 doi: 10.1101/gr.1239303 14597658 10.1101/gr.1239303 PMC403769

[27] Malnic B, Hirono J, Sato T, Buck LB (1999) Combinatorial receptor codes for odors. Cell 96: 713–723. 10089886 10.1016/s0092-8674(00)80581-4

[28] Nguyen D-Q, Webber C, Ponting CP (2006) Bias of selection on human copy-number variants. PLoS Genet 2(2): e20. 16482228 10.1371/journal.pgen.0020020 PMC1366494

[29] Young JM, Endicott RM, Parghi SS, Walker M, Kidd JM, et al (2008) Extensive copy-number variation of the human olfactory receptor gene family. Am J Hum Genet 83(2): 228–242. 18674749 10.1016/j.ajhg.2008.07.005 PMC2495065

[30] Niimura Y, Nei M (2003) Evolution of olfactory receptor genes in the human genome. Proc Natl Acad Sci USA 100: 12235–12240. 14507991 10.1073/pnas.1635157100 PMC218742

[31] Matsui A, Go Y, Niimura Y (2010) Degeneration of olfactory receptor gene repertories in primates: no direct link to full trichromatic vision. Mol Biol Evol 27: 1192–1200. 20061342 10.1093/molbev/msq003

[32] Olender T, Waszak SM, Viavant M, Khen M, Ben-Asher E, et al.. (2012) Personal receptor repertoires: olfaction as a model, BMC Genomics, 13: 414, 1–16. 10.1186/1471-2164-13-414 PMC3462693 22908908

[33] Zhang D, Qian Y, Akula N, Alliey-Rodriguez N, Tang J (2011) Bipolar Genome Study, Accuracy of CNV Detection from GWAS Data. PLoS One 13: 6 (1).. 10.1371/journal.pone.0014511 PMC3020939 21249187

[34] Conrad DF, Pinto D, Redon R, Feuk L, Gokcumen O, et al (2010) Origins and functional impact of copy number variation in the human genome. Nature 464: 704–712. 19812545 10.1038/nature08516 PMC3330748

[35] Kidd JM, Cooper GM, Donahue WF, Hayden HS, Sampas N, et al (2008) Mapping and sequencing of structural variation from eight human genomes. Nature 1: 453 (7191): 56–64. 10.1038/nature06862 PMC2424287 18451855

[36] Korn JM, Kuruvilla FG, McCarroll SA, Wysoker A, Nemesh J, et al (2008) Integrated genotype calling and association analysis of SNPs, common copy number polymorphisms and rare CNVs. Nat Genet 40: 1253–1260. 18776909 10.1038/ng.237 PMC2756534

[37] Nozawa M, Kawahara Y, Nei M (2007) Genomic drift and copy number variation of sensory receptor genes in humans. Proc Natl Acad Sci USA 104: 20421–20426. 18077390 10.1073/pnas.0709956104 PMC2154446

[38] Hasin Y, Olender T, Khen M, Gonzaga-Jauregui C, Kim PM, et al (2008) High-resolution copy-number variation map reflects human olfactory receptor diversity and evolution. PLoS Genet 4(11): e1000249. 18989455 10.1371/journal.pgen.1000249 PMC2570968

[39] Waszak SM, Hasin Y, Zichner T, Olender T, Keydar I, et al.. (2010) Systematic Inference of Copy-Number Genotypes from Personal Genome Sequencing Data Reveals Extensive Olfactory Receptor Gene Content Diversity. PLoS Comput Biol 6(11). 10.1371/journal.pcbi.1000988 PMC2978733 21085617

[40] Go Y, Niimura Y (2008) Similar Numbers but Different Repertoires of Olfactory Receptor Genes in Humans and Chimpanzees. Mol Biol Evol 25(9): 1897–1907. 18562338 10.1093/molbev/msn135

[41] Sharon D, Glusman G, Pilpel Y, Khen M, Gruetzner F, et al (1999) Primate evolution of an olfactory reeptor cluster: diversification by gene conversion and recent emergence of pseudogenes. Genomics 61(1): 24–36. 10512677 10.1006/geno.1999.5900

[42] Gilad Y, Segre D, Skorecki K, Nachman MW, Lancet D, et al (2000) Dichotomy of single-nucleotide polymorphism haplotypes in olfactory receptor genes and pseudogenes. Nat Genet 26: 221–224. 11017082 10.1038/79957

[43] Gilad Y, Przeworski M, Lancet D (2004) Loss of olfactory receptor genes coincides with the acquisition of full trichromatic vision in primates. PLoS Biol 2(1). 10.1371/journal.pbio.0020005 PMC314465 14737185

[44] Lekkerkerker CG, Boland JCh, et al (1962) Representation of a finite graph by a set of intervals on the real line. Fund Math 51: 45–64.

[45] Ngai J, Chess A, Dowling MM, Necles N, Macagno ER, et al (1993) Coding of olfactory information: topography of odorant receptor expression in the catfish olfactory epithelium. Cell 72: 667–680. 8453662 10.1016/0092-8674(93)90396-8

[46] Chess A, Simon I, Cedar H, Axel R (1994) Allelic inactivation regulates olfactory receptor gene expression. Cell 78: 823–834. 8087849 10.1016/s0092-8674(94)90562-2

