# Supplementary figures and images for: Unravelling the Complexity of Human Olfactory Receptor Repertoire by Copy Number Analysis across Population Using High Resolution Arrays
Source: PLoS One. 2013 Jul 3;8(7):e66843. doi: 10.1371/journal.pone.0066843 (PMC3700933; doi:10.1371/journal.pone.0066843)

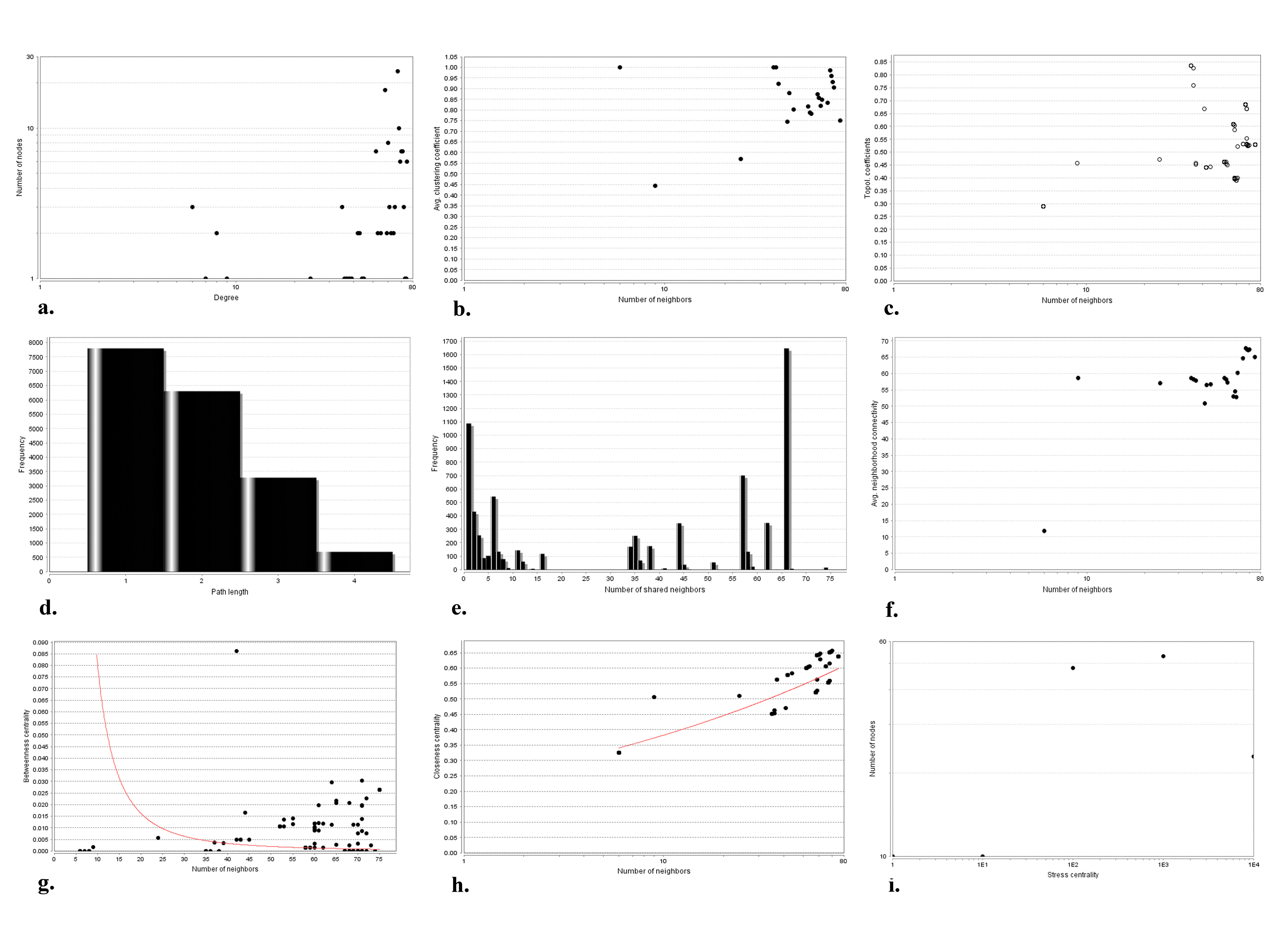

Supplement: Figure S1 — OR protein network containing 135 OR genes were analysed using NetworkAnalyzer considering it as an un-directed network. NetworkAnalyzer was also used to perform topological analysis containing both undirected and directed edges. (a) Displays the node degree of nodes (genes) having 80 edges linked to each, where in-degree distribution and out-degree distribution can be observed to distinguish between random and scale-free network OR topologies. (b) Displays the number of neighbors connectivity of node and is the number of its neighbors with respect to the average clustering coefficient. The neighborhood connectivity of a node n = 80 is the average connectivity of all neighbors of the entire OR gene network. (c) displays the number of neighbors connectivity of node and is the number of its neighbors with respect to the topological coefficient. The numerical attribute is clustered at 0.56 coefficient of n = 80 which should have a minimum of 2 neighbors to have a topological coefficient of zero. (d) shows the frequency of the nodes (OR genes) with respect to path length distribution. 4 path lengths across significantly varying frequency were observed for the edges between the OR gene nodes. (e) Number of shared neighbors for a given OR gene node can be seen here, with the large cluster containing ∼65 neighbors with a higher frequency followed by ∼1 neighbor with a much lower frequency. (f) Shows the n = 80 neighbors clustered at the average neighborhood connectivity of 60–70 neighbors each. (g) displays the betweenness centrality of all the neighbors in this network, the betweenness centrality value for each nodes is normalized by dividing by the number of node pairs shown along with the Fit of Power Law. (h) shows the closeness centrality of OR genes plotted against the number of OR gene neighbors with the reciprocal of the average shortest path length, the closeness centrality of each OR gene node is a number between 0 and 1 shown along with the Fitting line. (i) Dis [file pone.0066843.s001.tif]
