# Supplementary material for: Unravelling the Complexity of Human Olfactory Receptor Repertoire by Copy Number Analysis across Population Using High Resolution Arrays
Source: PLoS One. 2013 Jul 3;8(7):e66843. doi: 10.1371/journal.pone.0066843 (PMC3700933; doi:10.1371/journal.pone.0066843)
Supplement: Table S1 — Describes the −1 kb/+1 kb upstream and downstream sequences of the copy number breakpoint, 22,317,500 bp –22,474,268 bp and its homology within and across the genome of closely related ancestral species. These breakpoints help to understand the complexity of two distinct features of evolutionarily conserved and derived sequences of the genome. The columns following that denotes the upstream sequences (−1 kb) of breakpoint start region, followed by columns containing the percent of similarity. The other half of the Table shows regions of the upstream breakpoint that's has the highest identity hits in ancestral species and their % similarity. Ninth column shows the downstream region of the breakpoint end (+1 kb). Tenth and eleventh column contains other regions of breakpoint in humans and their % similarity. Twelfth and thirteenth column shows other sequence regions of downstream breakpoint region that have high sequence identity in ancestral species along with their % similarity. (DOC) [file pone.0066843.s002.doc]

| **Sl. No.** | **CNV Description ion** | **Upstream (-1)am (-1)** | **Hits**  **(Human)man)** | | **Identity**  **(**%**)** | | **Hit**  **(Ancestral)** | **Identity**  **(**%**)** | **( Downstream (+1)** | **Hits**  **(Human)(Human)3** | **Identity**  **(**%**)** | **Hit (Ancestral))5** | **Identity**  **(**%**)** |
| --- | --- | --- | --- | --- | --- | --- | --- | --- | --- | --- | --- | --- | --- |
| 1 | 22317500-22588019, 271, Chr 15q11.2 | 22316500-22317500 | RP11-2F9 | | 100 | | *Pan troglodytes,* Chr. 22,  CH251-302B8 | 98 | 22588019-22589019 | Chr. 15, RP11-603B24 | 100 |  | |
| 2 |  | 22316500-22317500 | Chr. 15,  RP11-350K22 | | 100 | | *Pongo abelii*,  Chr. 14,  CH276-454M15 | 96 | 22588019-22589019 | Chr. 15, CTD-2538I11 | 99 |  | |
| 3 |  | 22316500-22317500 | Chr. 15,  RP11-69H14 | | 100 | | *Pongo abelii,*  Chr. 12,  CH276-320G5 | 96 | 22588019-22589019 | Chr. 15, WI2-635I4 | 99 |  | |
| 4 |  | 22316500-22317500 | Chr. 15,  RP11-983G14 | | 99 | | *Rhesus Macaque* CH250-392A15 | 97 | 22588019-22589019 | Chr. 15,  ABC24-1588H5 | 99 |  | |
| 5 |  | 22316500-22317500 | Chr. 15, ABC12-7917949B4 | | 99 | |  |  | 22588019-22589019 | Chr. 15 COR2A-DD0002TASNU | 99 |  | |
| 6 |  | 22316500-22317500 | Chr. 15,  RP11-294C11 | | 99 | |  |  | 22588019-22589019 | Chr. 15, WI2-537I13 | 99 |  | |
| 7 |  | 22316500-22317500 | Chr. 15,  RP11-361C13 | | 99 | |  |  | 22588019-22589019 | RP11-5N22 | 94 |  | |
| 8 |  | 22316500-22317500 | Chr. 14  BAC R-597A11 | | 97 | |  |  | 22681064-22682064 | Chr. 15,  RP11-467N20 | 100 | *Pongo abelii,* Chr. 22,  CH276-381I22 | 100 |
| 9 |  | | | | | | | | 22681064-22682064 | Chr. 15,  CTD-3243P7 | 99 |  | |
| 10 |  | | | | | | | | 22681064-22682064 | Chr. 15,  CTD-2024P22 | 99 |  | |
| 11 |  | | | | | | | | 22681064-22682064 | Chr. 15, WI2-3073A15 | 99 |  | |
| 12 |  | | | | | | | | 22681064-22682064 | CH17-220P3 | 99 |  | |
| 13 |  | | | | | | | | 22681064-22682064 | Chr. 15, ABC14-1080422M2 | 99 |  | |
| 14 |  | | | | | | | | 22681064-22682064 | Chr. 15, ABC11-49393400N13 | 99 |  | |
| 15 |  | | | | | | | | 22681064-22682064 | Chr. 15, RP11-147B8 | 99 |  | |
| 16 |  | | | | | | | | 22681064-22682064 | CH17-184J24 | 99 |  | |
| 17 |  | | | | | | | | 22681064-22682064 | Chr. 15,  RP11-536P16 | 99 |  | |
| 18 |  | | | | | | | | 22681064-22682064 | Chr. 15, Pseudogene | 99 |  | |
| 19 |  | | | | | | | | 22681064-22682064 | Chr. 15,  RP11-483E23 | 99 |  | |
| 20 |  | | | | | | | | 22681064-22682064 | Chr. 13 | 100 |  | |
| 21 |  | | | | | | | | 22681064-22682064 | Chr. 8, RP11-10H3 | 96 |  |  |
| 22 |  | | | | | | | | 22494283-22495283 | Chr. 15,  RP11-603B24 | 100 |  |  |
| 23 |  | | | | | | | | 22494283-22495283 | Chr. 15, WI2-3656G8 | 99 |  |  |
| 24 |  | | | | | | | | 22494283-22495283 | Chr. 15,  RP11-361C13 | 98 |  |  |
| 25 |  | | | | | | | | 22494283-22495283 | Chr. 12,  RPCI11-792F18 | 95 |  |  |
| 26 |  | | | | | | | | 22494283-22495283 | Chr. 12, RCPI11-935C2 | 95 |  |  |
| 27 |  | | | | | | | | 22474268-22475268 | Chr. 15, RP11-960P22 | 100 | *Pan troglodytes,* Chr. 14,  CH251-419I2 | 97 |
| 28 |  | | | | | | | | 22474268-22475268 | Chr. 15, RP11-603B24 | 100 |  |  |
| 29 |  | | | | | | | | 22474268-22475268 | Chr. 15, RP11-361C13 | 99 |  |  |
| 30 |  | | | | | | | | 22474268-22475268 | ABC10-44509600J4 | 96 |  |  |
| 31 |  | | | | | | | | 22474268-22475268 | Chr. 17, CH17-224D4 | 96 |  |  |
| 32 |  | | | | | | | | 22474268-22475268 | Chr. 14, CH17-76I19 | 96 |  |  |
| 33 |  | | | | | | | | 22474268-22475268 | Chr. 14, ABC12-48925600G6 | 96 |  |  |
| 34 |  | | | | | | | | 22474268-22475268 | Chr. 14,  CH17-262H11 | 96 |  |  |
| 35 |  | | | | | | | | 22474268-22475268 | Chr. 14 | 96 |  |  |
| 36 | 20105479-20423360, 318,  Chr 14q11.2 | 20105479-20104479 | Chr. 14, R-244H18 | | | 100 | *Pan troglodytes,* rp43-26n14 | 98 | 20423360-20424360 | Chr. 14, 55G7 | 100 |  |  |
| 37 |  | | Chr. 22, ABC11-48289400I11 | | | 99 | *Pan troglodytes*, rp43-99g12 | 98 |  | Chr. 14, WI2-657F1 | 99 |  |  |
| 38 |  | | Chr. 14,  BAC R-254P21 | | | 99 | *Pan troglodytes*, Chr. 7, CH251-539J6 | 95 |  |  | |  |  |
| 39 |  | | Chr. 22 | | | 99 | *Pan troglodytes,* Chr. 7, CH251-321B16 | 95 |  |  | |  |  |
| 40 |  | | | Chr. 14, ABC7-42444800N20 | | 99 |  | | |  | |  |  |
| 41 |  | | Chr. 14, BAC R-597A11 | | | 99 |  | | |  | |  |  |
| 42 |  | | Chr. 7, RP1-42M2 | | | 95 |  | | | | | |  |
| 43 |  | | Chr. 7, RP1-42M2 | | | 95 |  | | | | |  | |
| 44 |  |  | Chr. 17 | | | 95 |  |  |  |  |  |  |  |
| 45 |  |  | Chr. 17, hRPK.215_P_18 | | | 95 |  |  |  |  |  |  |  |
| 46 | 19802529-20212323, 410, Chr 14q11.2 | 19801529-19802529 | Chr. 14, R-496I2 | | | 100 | *Pan troglodytes,* Chr. 22,  CH251-668I23 | 96 | 20212323-20213323 | Chr. 14, R-597A11 | 100 | Pongo abelii, Chr. 14, CH276-454M15 | 97 |
| 47 |  | | Chr. 14, ABC8-42110800I9 | | | 99 | *Pan troglodytes,* rp43-70b2 | 96 |  | ABC13-48065700I13 | 95 | Pongo abelii, Chr. 12, CH276-320G5 | 97 |
| 48 |  | | Chr. 14, ABC9-43894300J18 | | | 99 | *Pan troglodytes,* rp43-130h22 | 96 |  | Chr. 15, RP11-294C11 | 95 |  |  |
| 49 |  | | Chr. 14, R-491G9 | | | 99 | *Pan troglodytes,* Chr. 7, CH251-705K6 | 96 |  | Chr. 15, RP11-361C13 | 95 |  |  |
| 50 |  | | Chr. 14, R-536C10 | | | 99 | *Pan troglodytes,* Chr. 22, PTB-014E11 | 98 |  | | | | |
| 51 |  | | Chr. 14, R-597A11 | | | 99 | *Pan troglodytes,* Chr. X, CH251-432O16 | 96 |  | | | | |
| 52 |  | | Chr. 14, R-254P21 | | | 99 | *Pan troglodytes*, PTB-121B21 | 96 |  | | | | |
| 53 |  | | Chr. 22 | | | 99 | *Pan troglodytes,* Chr. X, CH251-549M16 | 95 |  | | | | |
| 54 |  | | Chr. 14, ABC12-49047100M9 | | | 99 | *Pan troglodytes,* Chr. X, CH251-660C21 | 95 |  | | | | |
| 55 |  | | RP11-11H9 | | | 95 | *Pan troglodytes,* Chr. X, CH251-161I7 | 95 |  | | | | |
| 56 |  | | Chr. 15,  RP11-983G14 | | | 95 | *Pan troglodytes,* Chr. 2, CH251-639G22 | 95 |  | | | | |
| 57 |  | | Chr. 15,  ABC12-7917949B4 | | | 95 | *Pongo abelii,* CH253-136E17 | 95 |  | | | | |
| 58 |  | | RP11-113C3 | | | 95 | *Pan troglodytes,* Chr. 14, CH251-680J1 | 98 |  | | | | |
| 59 |  | | Chr. 15,  RP11-294C11 | | | 95 | *Pongo abelii,* CH253-434E18 | 96 |  | | | | |
| 60 |  | | RP11-928F19 | | | 95 |  | | | | | | |
| 61 |  | | RP11-2F9 | | | 95 |  | | | | | | |
| 62 |  | | Chr. 15,  RP11-350K22 | | | 95 |  | | | | | | |
| 63 |  | | Chr. 15,  RP11-69H14 | | | 95 |  | | | | | | |
| 64 |  | | Chr. 15,  RP11-810K23 | | | 95 |  | | | | | | |
| 65 |  | | RP11-96F3 | | | 95 |  | | | | | | |
| 66 |  | | Chr. 12,  RP11-248E9 | | | 96 |  | | | | | | |
| 67 |  | | Chr. 3,  RP11-420J11 | | | 95 |  | | | | | | |
| 68 |  | | Chr. 14 | | | 95 |  | | | | | | |
| 69 |  | | Chr. 20,  RP5-873P14 | | | 100 |  | | | | | | |
| 70 |  | | Chr. 13 | | | 95 |  | | | | | | |
| 71 |  | | Chr. 11,  RP11-668M17 | | | 96 |  | | | | | | |
| 72 |  | | Chr. 11,  RP11-1084A14 | | | 96 |  | | | | | | |
| 73 |  | | Chr. 5,  CTD-2203A3 | | | 96 |  | | | | | | |
| 74 |  | | Chr. 11,  RP11-1023E15 | | | 96 |  | | | | | | |
| 75 |  | | ABC8-793122P21 | | | 96 |  | | | | | | |
| 76 |  | | Chr. 12,  RP11-324H9 | | | 96 |  | | | | | | |
| 77 |  | | RP11-453M23 | | | 96 |  | | | | | | |
| 78 |  | | Chr. 5,  RP11-373N22 | | | 96 |  | | | | | | |
| 79 |  | | Chr. 13,  RP11-513N16 | | | 96 |  | | | | | | |
| 80 |  | | Chr. 18,  RP11-765H19 | | | 96 |  | | | | | | |
| 81 |  | | Chr. 12,  RP11-18J10 | | | 96 |  | | | | | | |
| 82 |  | | Chr. X,  CH17-146C3 | | | 95 |  | | | | | | |
| 83 |  | | Chr. 11,  RP11-413N10 | | | 95 |  | | | | | | |
| 84 |  | | Chr. 11,  RP11-735P2 | | | 95 |  | | | | | | |
| 85 |  | | Chr. 11,  RP11-715M10 | | | 95 |  | | | | | | |
| 86 |  | | Chr. X,  RP13-137P7 on | | | 95 |  | | | | | | |
| 87 |  | | Chr. 5,  CTD-2020C3 | | | 95 |  | | | | | | |
| 88 |  | | Chr. 5, CTC-534K6 | | | 95 |  | | | | | | |
| 89 |  | | Chr. X, RP11-40I8 | | | 95 |  | | | | | | |
| 90 |  | | Chr. 4,  RP11-502A23 | | | 95 |  | | | | | | |
| 91 |  | | Chr. 1, RP11-449J1 | | | 95 |  | | | | | | |
| 92 |  | | Chr. X,  RP4-595A18 | | | 95 |  | | | | | | |
| 93 |  | | Chr. 6, RP1-162C6 | | | 95 |  | | | | | | |
| 94 |  | | Chr. 11,  PTB-147K15 | | | 95 |  | | | | | | |
| 95 |  | | Chr. 4,  RP11-173C9 | | | 95 |  | | | | | | |
| 96 |  | | Chr. 5,  CTD-2026D23 | | | 95 |  | | | | | | |
| 97 |  | | Chr. 5,  CTD-2170F21 | | | 95 |  | | | | | | |
| 98 |  | | Chr. 12,  RP11-185H22 | | | 95 |  | | | | | | |
| 99 |  | | Chr. 4,  RP11-312J17 | | | 95 |  | | | | | | |
| 100 |  | | Chr. 5, RP11-536I7 | | | 95 |  | | | | | | |
| 101 |  | | Chr. 13,  RP13-407F1 | | | 95 |  | | | | | | |
| 102 |  | | Chr. X,  RP11-22B10 | | | 95 |  | | | | | | |
| 103 |  | | Chr. X,  RP11-539N20 | | | 95 |  | | | | | | |
| 104 |  | | ABC14-50176700O13 | | | 95 |  | | | | | | |
| 105 |  | | Chr. 4 | | | 95 |  | | | | | | |
| 106 |  | | Chr. 4 | | | 95 |  | | | | | | |
| 107 |  | | Chr. 3 | | | 95 |  | | | | | | |
| 108 |  | | Chr. X | | | 95 |  | | | | | | |
| 109 |  | | Chr. X,  RP11-440N7 | | | 100 |  | | | | | | |
| 110 |  | | Chr. 16,  RP11-140O8 | | | 95 |  | | | | | | |
| 111 |  | | Chr. 3,  RP11-697B5 | | | 95 |  | | | | | | |
| 112 |  | | Chr. 18,  RP11-795F19 | | | 98 |  | | | | | | |
| 113 |  | | Chr. 18,  RP11-784B15 | | | 96 |  | | | | | | |
| 114 |  | | Chr. 19,  CTB-187L3 | | | 96 |  | | | | | | |
| 115 |  | | Chr. 3, RP11-521J5 | | | 95 |  | | | | | | |
| 116 |  | | Chr. 4,  RP11-592K15 | | | 95 |  | | | | | | |
| 117 |  | | Chr. X, LL0XNC01-157D4 | | | 95 |  | | | | | | |
| 118 |  | | Chr. 4,  RP11-371E22 | | | 95 |  | | | | | | |
| 119 |  | | Chr. 1,  RP11-240C17 | | | 95 |  | | | | | | |
| 120 |  | | Chr. 3, RP11-385J1 | | | 95 |  | | | | | | |
| 121 |  | | Chr. 2,  RP11-618J22 | | | 95 |  | | | | | | |
| 122 |  | | Chr. 1, RP11-95C20 | | | 97 |  | | | | | | |
| 123 |  | | Chr. 3,  RP11-663C11 | | | 98 |  | | | | | | |
| 124 |  | | Chr. 2,  CTD-2053I13 | | | 95 |  | | | | | | |
| 125 |  | | Chr. 11,  RP11-677I18 | | | 95 |  | | | | | | |
| 126 |  | | Chr. 5, CTC-222D7 | | | 95 |  | | | | | | |

**Table S1:** Describes the -1kb/+1kb upstream and downstream sequences of the copy number breakpoint, 22,317,500 bp - 22,474,268 bp and its homology within and across the genome of closely related ancestral species. These breakpoints helps to understand the complexity of two distinct features of evolutionarily conserved and derived sequences of the genome. The columns following that denotes the upstream sequences (-1kb) of breakpoint start region, followed by columns containing the percent of similarity. The other half of the Table shows regions of the upstream breakpoint that’s has the highest identity hits in ancestral species and their % similarity. Ninth column shows the downstream region of the breakpoint end (+1kb). Tenth and eleventh column contains other regions of breakpoint in humans and their % similarity. Twelfth and thirteenth column shows other sequence regions of downstream breakpoint region that have high sequence identity in ancestral species along with their % similarity.
