# Supplementary material for: Unravelling the Complexity of Human Olfactory Receptor Repertoire by Copy Number Analysis across Population Using High Resolution Arrays
Source: PLoS One. 2013 Jul 3;8(7):e66843. doi: 10.1371/journal.pone.0066843 (PMC3700933; doi:10.1371/journal.pone.0066843)
Supplement: Table S4 — Representation of the number of overlapping common Copy Number Polymorphisms (CNPs) found in the five populations studied including four from HapMap and Tibetan populations. CEU (CEPH collection), CHB (Han Chinese in Beijing, China), JPT (Japanese in Tokyo, Japan) and YRI (Yoruba in Ibadan, Nigeria), TBT (Tibetan). Y represents ‘Yes’ indicating the presence of that CNV in that particular population. (DOC) [file pone.0066843.s005.doc]

| Sl no  Table S4a: Representation of the number of overlapping common Copy Number Polymorphisms (CNPs) found in the five populations studied. Y= Yes. | Chromosome location | CNV Type | Start point | End point | Gene | CEU | CHB | JPT | YRI | IND |
| --- | --- | --- | --- | --- | --- | --- | --- | --- | --- | --- |
| 1 | 1q23.2 | Duplication | 159488058 | 159566647 | OR10j5 | - | - | - | - | Y |
| 2 | 1q23.2 | Deletion | 159343647 | 159438413 | OR10j1 | - | - | - | - | Y |
| 3 | 1q23.1 | Deletion | 158512618 | 158550080 | OR6y1  OR6p1  OR10x1 | - | - | - | - | Y |
| 4 | 5q35.3 | Duplication | 180115817 | 180216254 | OR2y1 | Y | - | - | - |  |
| 5 | 7q34 | Deletion | 141559739 | 141620280 | OR9a4 | - | - | - | - | Y |
| 6 | 7q35 | Duplication | 143655566 | 143688604 | OR2f1 | - | - | - | - | Y |
| 7 | 9q31.1 | Duplication | 105560931 | 107444557 | OR13f1  OR13c4  OR13c3  OR13c8  OR13c5  OR13c2  OR13c9 | - | - | - | Y | - |
| 8 | 9q33.2 | Duplication | 124233537 | 125560268 | OR1j1  OR1j2  OR1j4  OR1n1  OR1n2  OR1l8  OR1q1  OR1b1  OR1l3  OR1l4  OR1l6  OR5c1 | - | - | - | Y | - |
| 9 | 10q11.21 | Duplication | 45791083 | 45893408 | OR13a1 | - | - | - | - | Y |
| 10 | 11p11.2 | Deletion |  | 48968027 | OR2a47 | Y | - | - | - |  |
| 11 | 11p11.2 | Duplication | 48338872 |  | OR4c3  OR4c45 | - | - | - | - | Y |
| 12 | 11p15.4 | Deletion | 6790328 | 6829694 | OR2ag1  OR6a2 | - | - | - | - | Y |
| 13 | 11p15.4 | Duplication | 6837170 | 6881136 | OR10a5 | - | - | - | - | Y |
| 14 | 11p15.4 | Deletion | 6104041 | 6170921 | OR56b4 | - | - | - | - | Y |
| 15 | 11p15.4 | Deletion | 5544588 | 5575211 | OR52h1 | - | - | - | - | Y |
| 16 | 11p15.4 | Duplication | 4854937 | 4892742 | OR51s1 | - | - | - | - | Y |
| 17 | 11p15.4 | Duplication | 4676292 | 4737834 | OR51e1  OR51e2 | - | - | - | - | Y |
| 18 | 11p15.4 | Duplication | 5172397 | 5214368 | OR52a1 | - | - | - | - | Y |
| 19 | 11p15.4 | Duplication | 5448001 | 5546809 | OR51i1  OR51i2 | - | - | - | - | Y |
| 20 | 11p15.4 | Duplication | 4934100 | 4961558 | OR51g1  OR51g2 | - | - | - | - | Y |
| 21 | 11p15.4 | Deletion | 4629695 | 4671465 | OR51d1  OR51e1 | - | - | - | - | Y |
| 22 | 11p15.4 | Deletion | 5029456 | 5073101 | OR52j3 | - | - | - | - | Y |
| 23 | 11q11 | Deletion | 55437707 | 55608261 | OR5d13  OR5d14  OR5l1  OR5d18  OR5l2  OR5d16 | - | - | - | Y | - |
| 24 | 11q11 | Deletion | 55402800 | 55452996 | OR4p4  OR4s7  ORc6 | - | - | - | - | Y |
| 25 | 11q11 | Deletion | 55091267 | 55157853 | OR4a16  OR4a15 | - | - | - | - | Y |
| 26 | 11q11 | Deletion | 55363326 | 55460670 | OR4c11  OR4p4  OR4s2  OR4c6 | - | - | - | - | Y |
| 27 | 11q11 | Duplication | 55666486 | 55694807 | OR5w2 | - | - | - | - | Y |
| 28 | 11q11 | Deletion | 55074677 | 55146637 | OR4a16  OR4a15 | - | - | - | - | Y |
| 29 | 11q11 | Duplication | 55331281 | 55475789 | OR4c16  OR4c11  OR4p4  OR4s2  OR4c6 | - | - | - | - | Y |
| 30 | 11q24.1 | Deletion | 123777735 | 123819320 | OR8d4  OR4d5  OR6t1 | - | - | - | - | Y |
| 31 | 11q12.1 | Deletion | 57920450 | 57997078 | OR9q1  OR9q2  OR1s1  OR1s2  OR10q1 | - | - | - | - | Y |
| 32 | 11q12.1 | Deletion | 58237819 | 58334928 | OR5b21 | - | - | - | - | Y |
| 33 | 11q12.1 | Duplication | 56778757 | 56899223 | OR5ak4p | - | - | - | - | Y |
| 34 | 11q12.1 | Deletion | 56724009 | 56774386 | OR5ak2 | - | - | - | - | Y |
| 35 | 11q24.2 | Deletion | 12433948 | 124507019 | OR8b12  OR8a1 | - | - | - | - | Y |
| 36 | 12q13.2 | Duplication | 55790750 | 55891377 | OR6c76  OR6c2  OR6c70  OR6c65  OR6c68 | - | - | - | - | Y |
|  | 12q13.2 | Deletion | 55618259 | 55676365 | OR6c74 | - | - | - | - | Y |
|  | 12q13.2 | Deletion | 55703374 | 55769472 | OR6c1  OR6c3 | - | - | - | - | Y |
|  | 13q21.31 | Deletion | 64301592 | 64405310 | OR7e156p  OR4q3  OR4m1  OR4n2  OR4k2  OR4k5  OR4k1 | - | - | - | Y | - |
|  | 14q11.2 | Deletion | 19802529 | 20212323 | OR11h2 | Y | - | - | - | - |
|  | 14q11.1 | Duplication | 19068794 | 20434946 | OR11h2 | - | - | - | - | - |
|  | 14q11.1 | Duplication | 10916337 | 20400783 | OR11h2  OR4q3 | - | - | - | - | Y |
|  | 14q11.2 | Deletion | 19938429 | 20259951 | OR11h2  OR4q3  OR4m1  OR4n2  OR4k2  OR4k5  OR4k1 | Y | - | - | - | - |
|  | 14q11.2 | Duplication | 19437975 | 20413039 | OR11h2  OR4q3  OR4m1  OR4n2  OR4k2  OR4k5  OR4k1 | Y | - | - | - | - |
|  | 14q11.2 | Deletion | 19801473 | 20241852 | OR11h2  OR4q3 | - | - | - | - | - |
|  | 14q11.2 | Duplication | 20010901 | 20407630 | OR11h2  OR4q3  OR4m1  OR4n2  OR4k2  OR4k5  OR4k1 | - | - | - | Y | - |
|  | 14q11.2 | Duplication | 19844095 | 20383798 | OR11h2 | - | - | - | - | Y |
|  | 14q11.2 | Duplication | 19382197 | 20413846 | OR11h2 | - | - | - | - | Y |
|  | 14q11.2 | Duplication | 20203454 | 20425911 | OR4q3 | - | - | - | - | Y |
|  | 14q11.2 | Duplication and Deletion | 20315192 | 20422254 | OR4k1  OR4k5 | - | - | - | - | Y |
|  | 14q11.2 | Deletion | 20315192 | 20422254 | OR4k2 | - | - | - | - | Y |
|  | 14q11.2 | Deletion | 21081743 | 21203332 | OR4k17  OR6S1 | - | - | - | - | Y |
|  | 14q11.2 | Duplication | 19115283 | 19689305 | OR11h12 | - | - | - | - | Y |
|  | 14q11.2 | Deletion | 20568481 | 20610747 | OR4k17 | - | - | - | - | Y |
|  | 15q11.2 | Duplication | 20854501 | 22381399 | OR4m2 | - | Y | - | - | - |
|  | 15q11.2 | Duplication |  | 22521201 | OR4m2  OR4n3p | - | - | - | - | Y |
|  | 15q11.2 | Deletion | 21937313 | 22417351 | OR4m2  OR4n3p | - | - | - | - | Y |
|  |  | Duplication | 21937313 | 22417351 | OR4n4  OR4n3p | - | - | - | - | Y |
|  | 15q11.1 | Duplication | 20541968 | 22417541 | OR4m2 | - | - | Y | - | - |

| Sl no | Chromosome location | Start point | End point | CEU | CHB | JPT | YRI | TBT |
| --- | --- | --- | --- | --- | --- | --- | --- | --- |
| 1 | 1p36.33 | 61723 | 235716 | Y | - | - | Y | Y |
| 2 | 1p36.33 | 61723 | 229607 | Y | Y | - | Y | - |
| 3  Table S4b: Representationof the number of overlapping common Copy Number Polymorphisms (CNPs) found in the four HapMap and Tibetan populations studied, CEU (CEPH collection), CHB (Han Chinese in Beijing, China), JPT (Japanese in Tokyo, Japan) and YRI (Yoruba in Ibadan, Nigeria), TBT (Tibetan), Y represents Yes indicating the presence of that CNV in particular population. | 1p36.33 | 61723 | 564621 | Y | - | - | - | - |
| 4 | 1p36.33 | 61723 | 837073 | - | Y | Y | - | Y |
| 5 | 1p36.33 | 61723 | 229161 | - | - | Y | - |  |
| 6 | 1p36.33 | 61723 | 356530 | - | - | - | - | Y |
| 7 | 1q44 | 248683388 | 248810646 | - | - | - | Y | - |
| 8 | 1q44 | 247827956 | 248293158 | - | - | - | Y | - |
| 9 | 1q44 | 248688148 | 248790367 | - | - | - | Y | - |
| 10 | 1q44 | 248687790 | 248797201 | - | - | - | Y | - |
| 11 | 1q44 | 248641180 | 248797201 | - | - | - | - | Y |
| 12 | 1q44 | 248649488 | 248794396 | - | - | - | - | Y |
| 13 | 1q44 | 248683388 | 248882051 | - | - | - | - | - |
| 14 | 1q23.1 | 158530676 | 15858564 | - | - | - | - | - |
| 15 | 1q23.2 | 159488058 | 159566649 | - | - | - | - | Y |
| 16 | 2q37.3 | 240618118 | 241011075 | Y | - | - | - | - |
| 17 | 7q35 | 143917589 | 144040905 | Y | Y | - | Y | Y |
| 18 | 7q35 | 143917589 | 144066935 | Y | - | - | Y | - |
| 19 | 7q35 | 143917589 | 144033839 | - | - | - | - | Y |
| 20 | 7q35 | 143911612 | 144040905 | Y | - | - | - | - |
| 21 | 7q35 | 143911612 | 144066935 | - | - | - | Y | - |
| 22 | 7q35 | 143911612 | 144051219 | - | - | - | Y | - |
| 23 | 7q35 | 143927795 | 144051219 | - | - | - | Y | - |
| 24 | 7q35 | 143927795 | 144066935 | Y | - | - | Y | - |
| 25 | 7q35 | 143909223 | 144066935 | Y | - | - | - | - |
| 26 | 7q35 | 143909223 | 144027564 | - | - | - | - | Y |
| 27 | 7q35 | 143896240 | 144066935 | Y | Y | - | Y | - |
| 28 | 7q35 | 143896240 | 144006689 | Y | - | - | - | - |
| 29 | 7q35 | 143896240 | 144051219 | - | - | - | Y | - |
| 30 | 7q35 | 143896240 | 144040905 | - | - | - | Y | - |
| 31 | 7q35 | 143804670 | 144014216 | - | - | - | Y | - |
| 32 | 7q35 | 143896240 | 144027564 | - | - | - | Y | - |
| 33 | 7q35 | 143938301 | 144051219 | - | - | - | Y | - |
| 34 | 8p23.3 | 46386 | - | - | - | Y | - | Y |
| 35 | 11q11 | 55429395 | 55603568 | - | - | - | Y | - |
| 36 | 11q11 | 55429395 | 55603679 | - | - | - | Y | - |
| 37 | 11q11 | 55429395 | 55603457 | - | - | - | Y | - |
| 38 | 11q11 | 55689110 | 55826179 | - | - | - | Y | - |
| 39 | 11q11 | 55685799 | 55826179 | - | - | - | Y | - |
| 40 | 14q11.2 | 20105479 | 20423360 | Y | - | Y | Y | - |
| 41 | 14q11.2 | 19273613 | 20423360 | - | - | - | - | Y |
| 42 | 14q11.2 | 20200171 | 20423360 | Y | Y | Y | Y | - |
| 43 | 14q11.2 | 20200171 | 20420227 | Y | - | - | - | - |
| 44 | 14q11.2 | 20292138 | 20420227 | - | - | - | - | Y |
| 45 | 14q11.1 | 19002112 | - | Y | - | - | Y | - |
| 46 | 14q11.2 | 19562127 | 20423360 | - | Y | - | Y | - |
| 47 | 14q11.2 | 20105679 | 20423360 | - | Y | - | - | - |
| 48 | 14q11.2 | 19416179 | 20423360 | Y | - | Y | Y | - |
| 49 | 14q11.2 | 19562127 | 20392774 | Y | - | - | - | - |
| 50 | 14q11.2 | 20200171 | 20420277 | Y | - | - | - | - |
| 51 | 14q11.2 | 19697873 | 20423360 | - | Y | - | - | - |
| 52 | 14q11.2 | 19802592 | 20423360 | - | Y | - | - | - |
| 53 | 14q11.2 | 19689309 | 20420227 | - | Y | - | - | - |
| 54 | 14q11.2 | 20107459 | 20423360 | - | Y | - | - | - |
| 55 | 14q11.2 | 19562127 | 20420227 | - | - | Y | - | - |
| 56 | 14q11.2 | 19620039 | 20423360 | - | - | Y | - | - |
| 57 | 14q11.2 | 19801743 | 20423360 | - | - | Y | - | - |
| 58 | 14q11.2 | 20105479 | 20413846 | - | - | - | Y | - |
| 59 | 14q11.2 | 20226769 | 20413846 | - | - | - | - | Y |
| 60 | 14q11.2 | 19453314 | 20423360 | - | - | - | Y | - |
| 61 | 14q11.2 | 19115283 | 19453314 | - | - | - | - | Y |
| 62 | 14q11.2 | 19335739 | - | - | - | - | - | Y |
| 63 | 14q11.2 | 19562127 | 20425583 | - | - | - | Y | - |
| 64 | 15q11.2 | 20216943 | 22588019 | Y | Y | Y | Y | - |
| 65 | 15q11.2 | 22317500 | 22588019 | Y | Y | Y | Y | Y |
| 66 | 15q11.2 | 22317500 | 22494283 | - | - | - | - | Y |
| 67 | 15q11.2 | 22317500 | 22474268 | - | - | - | - | Y |
| 68 | 15q11.2 | 22280465 | 22673387 | Y | - | - | - | - |
| 69 | 15q11.2 | 20870339 | 22673387 | - | - | - | - | Y |
| 70 | 15q11.2 | 22301994 | 22681064 | Y | - | - | Y | Y |
| 71 | 15q11.2 | 22301994 | 22673387 | - | - | - | - | Y |
| 72 | 15q11.2 | 22280465 | 22681064 | Y | - | - | Y | - |
| 73 | 15q11.2 | 22301994 | 22588019 | Y | Y | Y | Y | Y |
| 74 | 15q11.2 | 22280465 | 22588019 | Y | Y | - | Y | - |
| 75 | 15q11.2 | 22384989 | 22588019 | Y | - | - | - | - |
| 76 | 15q11.2 | 21905745 | 22681064 | Y | - | - | - | - |
| 77 | 15q11.2 | 21929986 | 22681064 | - | - | - | - | Y |
| 78 | 15q11.2 | 20853080 | 22681064 | Y | - | - | - | - |
| 79 | 15q11.2 | 21905745 | 22588019 | Y | - | Y | - | - |
| 80 | 15q11.2 | 22317500 | 22681064 | Y | - | - | - | - |
| 81 | 15q11.2 | 22396241 | 22588019 | Y | - | - | - | - |
| 82 | 15q11.2 | 22384989 | 22681064 | Y | - | - | - | - |
| 83 | 15q11.2 | 20472839 | 22588019 | - | Y | - | - | - |
| 84 | 15q11.2 | 21271038 | 22588019 | - | Y | Y | Y | - |
| 85 | 15q11.2 | 22042506 | 22588019 | - | Y | Y | Y | - |
| 86 | 15q11.2 | 20806345 | 22588019 | - | - | Y | - | - |
| 87 | 15q11.2 | 21069837 | 22588019 | - | - | Y | - | - |
| 88 | 15q11.2 | 20440526 | 22681064 | - | - | Y | - | - |
| 89 | 15q11.2 | 22042506 | 22681064 | - | - | Y | - | - |
| 90 | 15q11.2 | 22301994 | 23282799 | - | - | - | Y | - |
| 91 | 15q11.2 | 21284264 | 22588019 | - | - | - | Y | - |
| 92 | 15q11.1 | 20284054 | 22588019 | - | Y | - | Y | - |
| 93 | 15q11.2 | 22301994 | 22456092 | - | - | - | Y | - |
| 94 | 15q11.2 | 21238972 | 22384989 | - | - | - | Y | - |
| 95 | 15q11.2 | 21919572 | 22588019 | - | - | - | - | - |
| 96 | 15q11.2 | 22280465 | 22422348 | - | - | - | - | - |
| 97 | 15q11.1 | 21271038 | 22474588 | Y | - | - | - | - |
| 98 | 15q11.1 | 22338943 | 22474588 | - | - | - | - | Y |
| 99 | 15q11.1 | 20590015 | 22588019 | Y | - | - | - | - |
| 100 | 15q11.1 | 20262224 | 20263387 | Y | - | - | - | - |
| 101 | 15q11.1 | 20395517 | 22588019 | Y | Y | - | Y | - |
| 102 | 15q11.1 | 20395517 | 22396241 | - | - | - | - | Y |
| 103 | 15q11.1 | 20262224 | 22588019 | Y | - | - | Y | - |
| 104 | 15q11.1 | 20590015 | 22388224 | Y | - | - | - | - |
| 105 | 15q11.1 | 20216943 | 22861064 | Y | - | - | Y | - |
| 106 | 15q11.1 | 20601542 | 22588019 | Y | - | - | - | - |
| 107 | 15q11.1 | 20532605 | 22422348 | Y | - | - | - | - |
| 108 | 15q11.1 | 20262224 | 22681064 | Y | - | - | - | - |
| 109 | 15q11.1 | 20472839 | 22681064 | Y | - | - | - | - |
| 110 | 15q11.1 | 20556430 | 22588019 | - | Y | - | - | - |
| 111 | 15q11.1 | 20291301 | 22588019 | - | Y | - | - | - |
| 112 | 15q11.1 | 20440526 | 22588019 | - | - | Y | - | - |
| 113 | 15q11.1 | 20590015 | 22861064 | - | - | Y | - | - |
| 114 | 15q11.1 | 20203949 | 22861064 | - | - | Y | - | - |
| 115 | 15q11.1 | 20590015 | 23487534 | - | - | - | Y | - |
| 116 | 15q11.1 | 20216943 | 22588019 | - | - | - | Y | - |
| 117 | 15q11.1 | 20446126 | 22588019 | - | - | - | Y | - |
| 118 | 15q11.1 | 20488324 | 22588019 | - | - | - | Y | - |
| 119 | 15q11.1 | 22398905 | 22588019 | - | - | - | - | Y |
| 120 | 15q11.1 | 20446126 | 22673387 | - | - | - | Y | - |
| 121 | 15q11.1 | 20585976 | 22464210 | - | - | - | - | Y |
| 122 | 15q11.1 | 20585976 | 22456092 | - | - | - | - | Y |
