# Supplementary material for: Unravelling the Complexity of Human Olfactory Receptor Repertoire by Copy Number Analysis across Population Using High Resolution Arrays
Source: PLoS One. 2013 Jul 3;8(7):e66843. doi: 10.1371/journal.pone.0066843 (PMC3700933; doi:10.1371/journal.pone.0066843)
Supplement: Table S5 — Hotspot analysis using HD-CNV of the CNPs was used to identify the hotspots in chromosomes of various populations. (DOC) [file pone.0066843.s006.doc]

Table S5: Hotspot analysis using HD-CNV of the CNPs was used to identify the hotspots in chromosomes of various populations.

| Chromosome | Hotspot | Intermediate | Rare |
| --- | --- | --- | --- |
| 1 | 61 | 64 | 8 |
| 2 | 2 | 2 | 2 |
| 6 | 4 | 0 | 2 |
| 7 | 370 | 0 | 4 |
| 8 | 6 | 6 | 6 |
| 9 | 14 | 0 | 7 |
| 11 | 45 | 19 | 42 |
| 12 | 5 | 3 | 1 |
| 13 | 7 | 7 | 7 |
| 14 | 401 | 11 | 9 |
| 15 | 384 | 40 | 4 |
| 19 | 3 | 3 | 3 |
| 22 | 5 | 5 | 5 |
